# Rapamycin promotes differentiation increasing βIII-tubulin, NeuN, and NeuroD while suppressing nestin expression in glioblastoma cells

**DOI:** 10.18632/oncotarget.15906

**Published:** 2017-03-18

**Authors:** Michela Ferrucci, Francesca Biagioni, Paola Lenzi, Stefano Gambardella, Rosangela Ferese, Maria Teresa Calierno, Alessandra Falleni, Alfonso Grimaldi, Alessandro Frati, Vincenzo Esposito, Cristina Limatola, Francesco Fornai

**Affiliations:** ^1^ Department of Translational Research and New Technologies in Medicine and Surgery, Human Anatomy, University of Pisa, Pisa, Italy; ^2^ Istituto di Ricovero e Cura a Carattere Scientifico, Neuromed, Pozzilli, Isernia, Italy; ^3^ Department of Clinical and Experimental Medicine, University of Pisa, Pisa, Italy; ^4^ Department of Physiology and Pharmacology, La Sapienza University of Rome, Roma, Italy

**Keywords:** mammalian target of rapamycin, stem cells, neuronal differentiation, transmission electron microscopy, qRT-PCR, Gerotarget

## Abstract

Glioblastoma cells feature mammalian target of rapamycin (mTOR) up-regulation which relates to a variety of effects such as: lower survival, higher infiltration, high stemness and radio- and chemo-resistance. Recently, it was demonstrated that mTOR may produce a gene shift leading to altered protein expression. Therefore, in the present study we administered different doses of the mTOR inhibitor rapamycin to explore whether the transcription of specific genes are modified. By using a variety of methods we demonstrate that rapamycin stimulates gene transcription related to neuronal differentiation while inhibiting stemness related genes such as nestin. In these experimental conditions, cell phenotype shifts towards a pyramidal neuron-like shape owing long branches. Rapamycin suppressed cell migration when exposed to fetal bovine serum (FBS) while increasing the cell adhesion protein phospho-FAK (pFAK). The present study improves our awareness of basic mechanisms which relate mTOR activity to the biology of glioblastoma cells. These findings apply to a variety of effects which can be induced by mTOR regulation in the brain. In fact, the ability to promote neuronal differentiation might be viewed as a novel therapeutic pathway to approach neuronal regeneration.

## INTRODUCTION

Glioblastoma multiforme (GBM, grade IV astrocytoma) is the most common and highly malignant brain tumor, which intensely proliferates, infiltrates, and produces relapses early, during its progression [1, 2].

Among various molecular hallmarks, GBM cells are characterized by up-regulation of the molecular complex known as mammalian target of rapamycin (mTOR). In particular, mTOR up-regulation is related to lower survival [3, 4], higher infiltration [5–7], higher amount of stem cells [8–13], and higher resistance to radio- [7, 14–16] and chemo-therapy [17].

Despite mTOR is pivotal in regulating cell metabolism by acting on a number of cell functions [18–20], its activity is strongly related to the inhibition of the autophagy pathway. In fact, autophagy suppression represents another hallmark of GBM cells as demonstrated by using a variety of experimental approaches [21, 22].

In previous studies we demonstrated that the mTOR inhibitor rapamycin suppresses the growth of GBM both *in vivo* in mouse brain xenograft as well as *in vitro* both in cell lines and in patient-derived cell cultures. Previous studies we co-authored, evidenced by cytofluorimetry that these effects in GBM cells are associated with inhibition of cell growth and suppression of cell migration rather than a frank cytotoxicity [5, 23]. In a recent manuscript it was demonstrated that mTOR inhibition as well as temozolomide may produce a phenotypic shift led by gene modulation and altered protein expression [24, 25]. These phenotypic changes were related to cell proliferation, colony formation and migration and can be reproduced by rapamycin-induced altered gene expression. Therefore, in the present study we administered different doses of the mTOR inhibitor rapamycin to explore whether a dose-response variation in the transcription of specific genes was induced concomitantly with a wide range of phenotypic variations which were never simultaneously explored so far. These variations encompass cell number, gross cell morphology, the amount and the length of newly developed cell branches, the variations in the expression of the stem-like protein nestin as well as early mitotic (βIII-tubulin and NeuroD) and late post-mitotic (NeuN) neuronal markers and the glial fibrillary acidic protein (GFAP). The expression of these proteins was measured by using immunohistochemistry as well as immunoelectronmicroscopy and SDS-Page immune-blotting. The pattern of protein expression was backed up by measuring transcripts by quantitative real time- polymerase chain reaction (qRT-PCR). These phenotypic changes induced by increasing doses of rapamycin were correlated with suppression of mTOR activity (dose-dependent decrease of p6S) and inhibition of cell migration, which was further related to the expression of the migration-related adhesion protein phospho-FAK (pFAK). All these findings occurred consistently along three different GBM cell lines with only slight variations in the dose-response curves.

## RESULTS

### Effects of low doses of rapamycin on the number of U87MG cells

**Figure 1:**
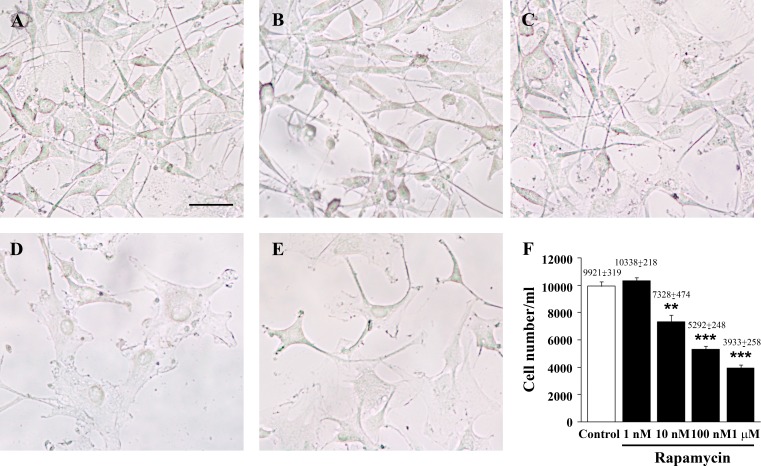
Rapamycin dose-dependently reduces the number of U87MG cells Representative pictures of non-fixed, non-stained U87MG cells treated either with vehicle (control) **A**. or rapamycin (1 nM, **B**. 10 nM, **C**. 100 nM, **D**. 1 μM, **E**.) for 24 h. The graph reports the total number of cells counted in 1 ml by using the Bürker chamber **F**. Values are given as the mean±S.E.M. Comparisons between groups were made by using one-way ANOVA with Scheffé post-hoc test. **P ≤ 0.05 *vs* control and 1 nM rapamycin. ***P ≤ 0.05 *vs* control and rapamycin at 1 nM and 10 nM. Scale bar = 27 μm.

In U87MG cells increasing doses of rapamycin, from 1 nM up to 1 μM for 24 h, were administered to produce increasing inhibition of mTOR. Rapamycin exposure decreases the number of cells, which is significant at the dose of 10 nM, and progresses at the doses of 100 nM and 1 μM (Figure 1). This reduction in cell number was not dependent on cell death. In fact, when we counted the number of trypan blue-stained cells, no significant difference was found for any dose of rapamycin used compared with baseline conditions (Figure 1F). This is in line with what we published previously [23], when we demonstrated, by using cytofluorimetry that in U87MG and GBM patient cells, a short-time treatment of rapamycin arrests cell proliferation. Autophagy and apoptotic cell death could be observed only in a few cells when rapamycin was administered for longer times at very high doses. Similarly, when tested in other cell lines, the very same doses of rapamycin produced a decrease in the number of U251MG (Supplementary Figure 1) and A172 cells (Supplementary Figure 2) which was significant at 1 μM and 100 nM, respectively.

### Effects of low doses of rapamycin on the U87MG cell morphometry

**Figure 2:**
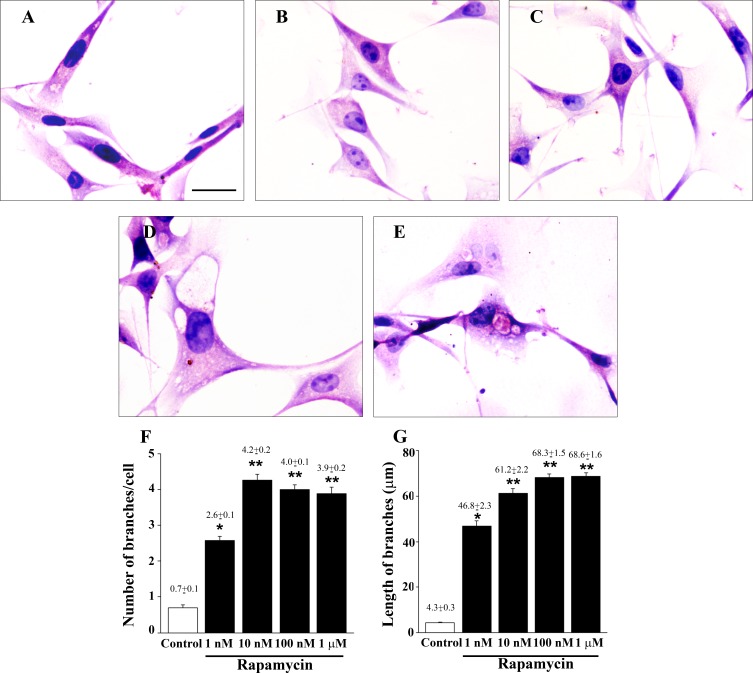
Rapamycin dose-dependently modifies cell morphology and cell branches Representative pictures of H&E-stained U87MG cells. The fusiform cell body observed in control cells **A**. disappears in cells exposed to increasing doses of rapamycin for 24 h **B**.-**E**. Rapamycin produces a dose-dependent increase in the diameter of the cell body which develops a pyramidal shape. 1 nM (B); 10 nM (C); 100 nM (D); 1 μM (E) of rapamycin exposure. These dose-dependent changes in the shape of the cell body are associated with a dose-dependent increase both in the number and length of cell branching, as reported in the graphs (**F**. and **G**., respectively). Values are given as the mean±S.E.M. Comparisons between groups are made by using one-way ANOVA with Scheffé post-hoc test. *P ≤ 0.05 *vs* control. **P ≤ 0.05 *vs* control and 1 nM rapamycin. Scale bar = 18 μm.

Exposure to increasing doses of rapamycin produced dose-dependently morphological changes. In fact, the typical fusiform cell body, observed in control cells (Figure 2A), disappears and rapamycin-treated cells develop, dose-dependently, a neuron-like pyramidal cell body with increased branching (Figures 2B-2E). These changes occur along with an increase both in number and length of cell branches (Figure 2). In detail, in control cells we counted 0.7±0.1 branches per cell, whereas in rapamycin-treated cells the amount of branches increases up to 4.2±0.2 reaching a plateau at the dose of 10 nM, (Figure 2F). In rapamycin-treated cells the increase in the number of cell branching was counted concomitantly with the measurement of increasing length of each branch (Figure 2G). In fact, while in control cells the mean length per branch was 4.3±0.3 μm, following 1 nM rapamycin the length increases to 46.8±2.3 μm. The branch lenght further increases dose-dependently to reach a plateau: 61.2±2.2 μm at 10 nM, and 68.3±1.5 and 68.6±1.6 μm at 100 nM and 1 μM of rapamycin, respectively (Figure 2G). Remarkably, the amount of the effects produced from each dose of rapamycin (above 10 nM) was underestimated compared with controls since we missed on purpose those branches measuring length above 300 μM. This was done to keep the measurement within a range of length allowing to visualize the branch reliably along its course in the same focal plane. Indeed following rapamycin a few branches surpassed the length of 300 μM, although no difference in their number was counted between rapamycin doses above 10 nM leaving intact the differences between groups for these high rapamycin doses. A dose-dependent increase in the length of cell branching was also observed in rapamycin-treated U251MG (Supplementary Figure 3) and A172 cells (Supplementary Figure 4), whereas in neither these cell lines rapamycin was able to induce an increase in the number of cell branches (Supplementary Figures 3 and 4).

### The stemness antigen nestin is suppressed dose-dependently by rapamycin

**Figure 3:**
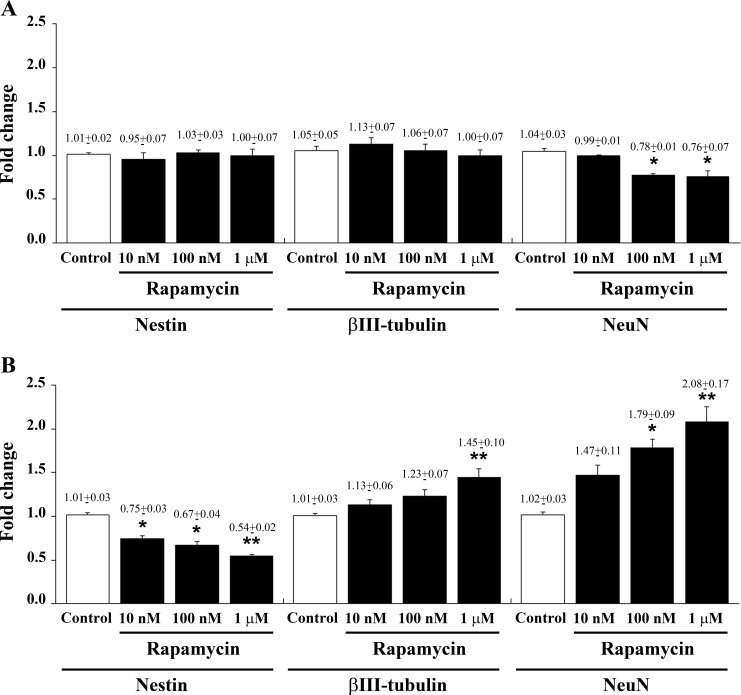
Rapamycin dose-dependently reduces nestin while increasing βIII-tubulin and NeuN mRNAs Specific gene expression was assessed by quantitative real-time PCR analysis (means from two different experiments normalized with two different internal references) for nestin, βIII-tubulin and NeuN in U87MG cells in control conditions and following 4 h **A**. and 24 h **B**. of 10 nM, 100 nM and 1 μM rapamycin. Data are expressed as the means±SEM. Comparison between groups are made by using one-way ANOVA with Bonferroni test. *P ≤ 0.05 *vs* respective control. **P ≤ 0.05 *vs* respective control and 10 nM rapamycin.

**Figure 4:**
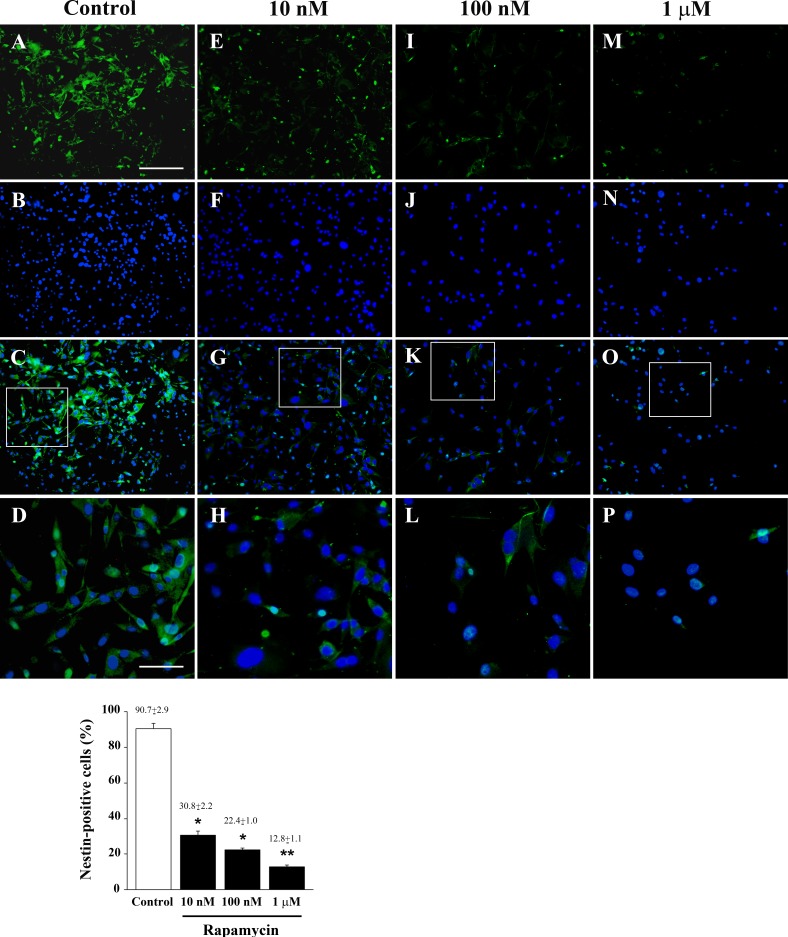
Low doses of rapamycin dose-dependently reduce nestin in U87MG cells Immune-fluorescence of U87MG cells treated with vehicle **A**.-**D**. and rapamycin at the dose of 10 nM **E**.-**H**. 100 nM **I**.-**L**. 1 μM **M**.-**P**. In the first line cells were stained for the stem cell marker nestin. Rapamycin produces a dose-dependent decrease of nestin immune-fluorescence. In the second line cells were stained for the nuclear dye DAPI. In the third line the merging between nestin (green) and DAPI (blue) fluorescence is shown. In the fourth line, a high magnification of the squared insert of line three is shown. The graph reports the percentage of nestin-positive cells in control and after treatment with different doses of rapamycin. Values are given as the mean±S.E.M. Comparisons between groups are made by using one-way ANOVA with Scheffé post-hoc test. *P ≤ 0.05 *vs* control. **P ≤ 0.05 *vs* control and 10 nM rapamycin. Scale bars = (A-C, E-G, I-K, M-O) 155 μm; (D, H, L, P) 45 μm.

Despite the transcript of nestin was not affected at 4 h following each dose of rapamycin (Figure 3A), qRT-PCR carried out at 24 h following rapamycin administration indicates a dose-dependent decrease of the stem cell marker nestin (Figure 3B). As reported for morphological changes in the previous paragraph, we documented a remarkable decrease (almost 50%) in nestin which is already significant for the dose of 10 nM of rapamycin. Higher doses of rapamycin further decrease only slightly nestin transcript levels. The reduction of nestin mRNA produced by rapamycin was consistent independently from the transcript used as internal reference, as detailed in Supplementary Figure 5 (which reports the consistency of quantitative data for each mRNA along with two different ratio for the internal reference mRNA, actin or globin). A progressive dose-dependent decrease of nestin mRNA was also observed in U251MG and A172 cells at 24 h after rapamycin administration (Supplementary Figure 6). Immune-fluorescence for nestin overlaps with qRT PCR data showing showing a remarkable decrease already for the lowest dose of rapamycin. Only the highest rapamycin dose produced a further significant reduction in nestin (Figure 4 graph). In representative pictures of Figure 4 it is evident the intense nestin immune-fluorescence in control conditions, which markedly decreases dose-dependently under the effects of rapamycin. The loss of cell staining strikingly contrasts with preservation of the number of cells as represented in the second line by DAPI immune-fluorescence and by merging pictures reported in the third line. The lowest line provides a higher magnification which allows to appreciate how the reduction in nestin is the consequence of both a decrease in the number of immune-positive cells and the decrease of stainable antigens within each cell. As a side observation, in these pictures one can appreciate the wide nuclear volume produced by the highest doses of rapamycin as evidenced by DAPI fluorescence which confirms the morphological changes reported with Haematoxylin & Eosin (H&E) staining of Figure 2.

Similar findings were obtained in U251MG and A172 cells after increasing doses of rapamycin, as shown in Supplementary Figures 7 and 8, respectively.

**Figure 5:**
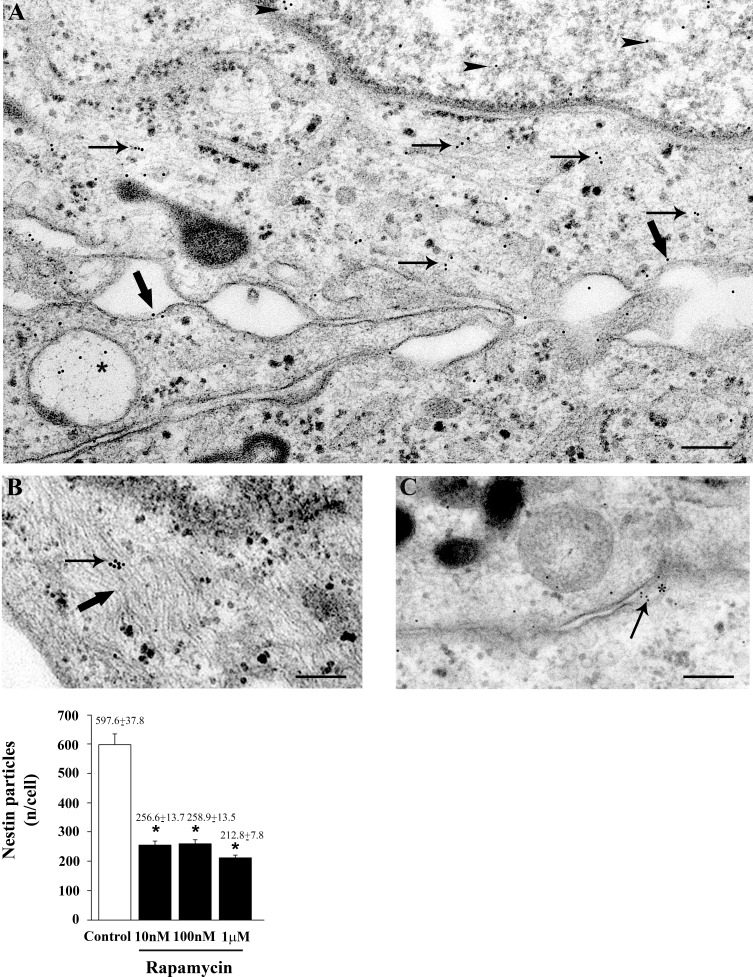
Rapamycin dose-dependently reduces nestin immune-cytochemistry Representative pictures of immune-cytochemistry for nestin in control cells (A-C). Nestin immune-gold particles are localized in the nucleus (arrowheads) and cytoplasm (arrows), over/under the plasma membrane (thick arrows) and they are placed with autophagy-like vacuoles (*) **A**. Nestin immune-gold particles (arrow) are present on cytoplasmic filaments (thick arrow) **B**. and in a narrow space between two cells which appear to be connected by a bridge of the plasma membrane (*) **C**. The graph reports the rapamycin-induced dose-dependent decrease in the counts of nestin immune-gold particles (10 nM; 100 nM and 1 μM). Values are given as the mean±S.E.M. Comparisons between groups are made by using one-way ANOVA with Scheffé post-hoc test. *P ≤ 0.05 *vs* control. Scale bars= (A, B) = 0.19 μm; (C) = 0.22 μm.

The subcellular placement of nestin can be appreciated following representative immune-electron microscopy of Figures 5A-5C, where nestin-bound immune-gold particles are placed both in the nucleus and cytoplasm, where they are more abundant within autophagy-like vacuoles and over the cytoplasmic filaments lining close to plasma membrane. The amount of nestin particles counted stoichiometrically through immune-gold particles are reduced dose-dependently already for the dose of 10 nM. Similar results were obtained when nestin immune-gold particles were analyzed in U251MG and A172 cells, as shown in representative pictures and graphs of Supplementary Figures 9 and 10, respectively.

**Figure 6:**
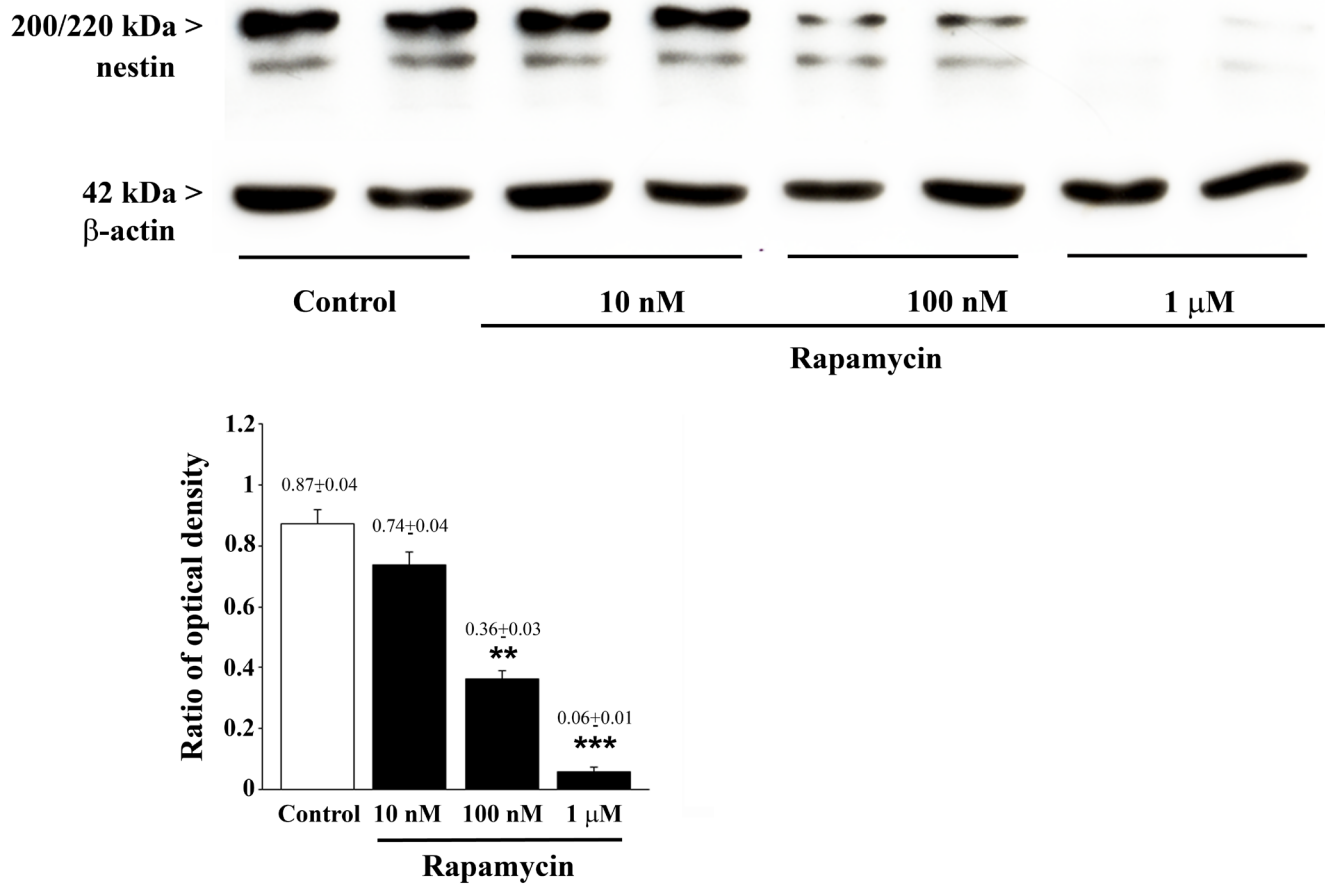
Rapamycin dose-dependently reduces nestin assessed by immune-blotting Representative immune-blots for nestin and the housekeeping protein β-actin in control and rapamycin-treated U87MG cells. The ratio between the optical densities of nestin and β-actin is reported in the graph. Rapamycin reduces nestin dose-dependently. Values are given as the mean±S.E.M. Comparisons between groups were made by using one-way ANOVA with Bonferroni test. **P ≤ 0.05 *vs* control and 10 nM rapamycin. ***P ≤ 0.05 *vs* control and rapamycin at 10 nM and 100 nM.

The semi-quantitative measurement based on optical density of the ratio of immune-positive bands of nestin vs β-actin in U87MG cells (Figure 6) reproduces rapamycin-induced suppression established by other methods, although slight differences are noticeable for increasing rapamycin doses. These are likely to rely on the lower detection sensitivity and specificity of immune-blotting compared with quantitative methods reported above.

### Rapamycin dose-dependently increases βIII-tubulin

**Figure 7:**
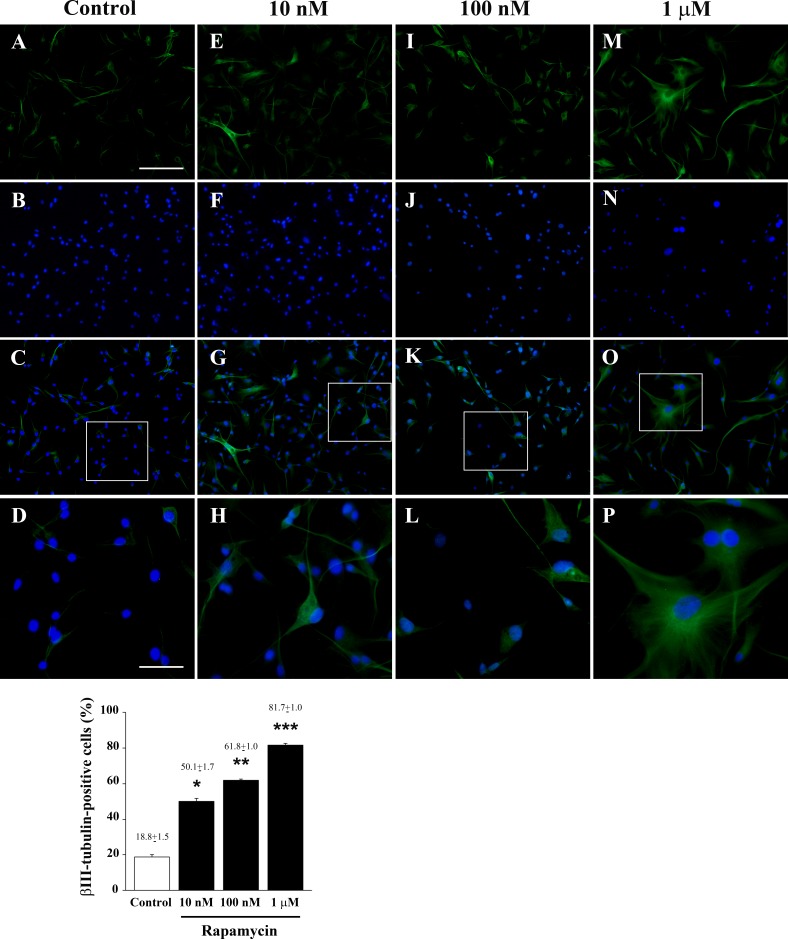
Rapamycin dose-dependently increases βIII-tubulin immune-fluorescence Immune-fluorescence of U87MG cells treated with vehicle **A**.-**D**. and rapamycin at the dose of 10 nM **E**.-**H**. 100 nM **I**.-**L**. 1 μM **M**.-**P**. In the first line cells were stained for the early neuronal marker βIII-tubulin. Rapamycin increases dose-dependently immune-fluorescence. In the second line cells were stained for the nuclear dye DAPI. In the third line the merging between βIII-tubulin (green) and DAPI (blue) fluorescence is shown. In the fourth line a high magnification of the squared insert of line three is shown. The graph reports the percentage of βIII-tubulin-positive cells in control and after treatment with different doses of rapamycin. Values are given as the mean±S.E.M. Comparisons between groups were made by using one-way ANOVA with Scheffé post-hoc test. *P ≤ 0.05 *vs* control. **P ≤ 0.05 *vs* control and 10 nM rapamycin. ***P ≤ 0.05 *vs* control and rapamycin at 10 nM and 100 nM. Scale bars = (A-C, E-G, I-K, M-O) 155 μm; (D, H, L, P) 45 μm.

**Figure 8:**
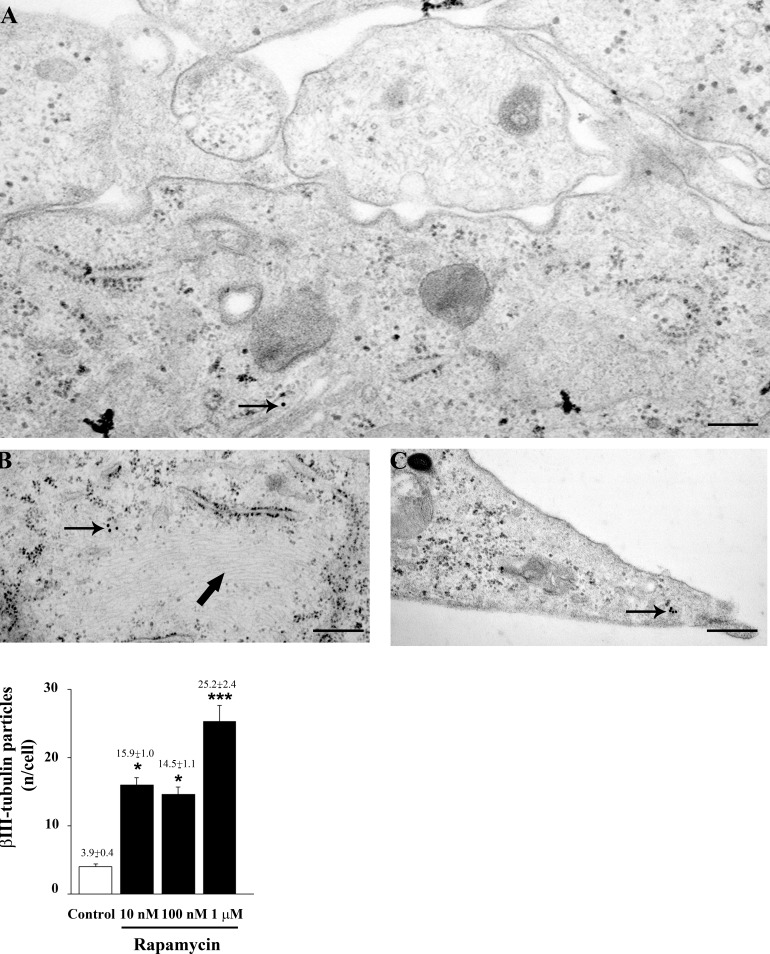
Rapamycin increases βIII-tubulin immune-cytochemistry In representative micrograph **A**. scanty immune-gold particles for βIII-tubulin (arrow) are shown in control cells. After rapamycin immune-gold particles increase (arrow) and they are placed close to cytoplasmic filaments (thick arrows) **B**. and within cell branches **C**. The graph reports the dose-dependent increase in βIII-tubulin immune-gold particles induced by rapamycin (10 nM; 100 nM and 1 μM). Values are given as the mean±S.E.M. Comparisons between groups were made by using one-way ANOVA with Scheffé post-hoc test. *P ≤ 0.05 *vs* control. ***P ≤ 0.05 *vs* control and rapamycin at 10 nM and 100 nM. Scale bars= (A) = 0.23 μm; (B) = 0.37 μm; (C) = 0.33 μm.

**Figure 9:**
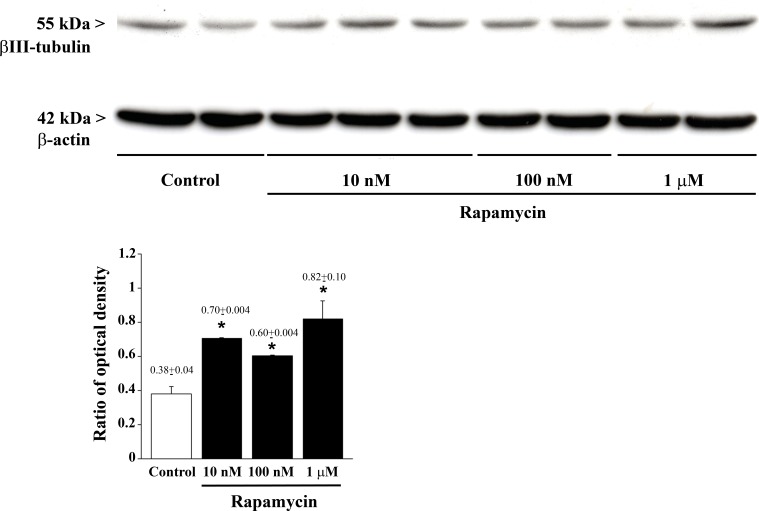
Rapamycin dose-dependently increases βIII-tubulin assessed by immune-blotting Representative immune-blotting showing βIII-tubulin and the housekeeping protein β-actin in control and rapamycin-treated U87MG cells. The graph report the βIII-tubulin/β-actin ratios between optical densities showing that rapamycin increases dose-dependently βIII-tubulin. Values are given as the mean±S.E.M. Comparisons between groups were made by using one-way ANOVA with Bonferroni test. *P ≤ 0.05 *vs* control.

As reported in Figure 3, rapamycin produces an increase in the mRNA for βIII-tubulin at 24 h but not at 4 h of exposure. This increase in βIII-tubulin occurs harmonically for various doses of rapamycin. Thus, the highest rapamycin dose still produces a significant increase compared with other doses of rapamycin (Figure 3B). A similar dose-dependent increase in the mRNA for βIII-tubulin at 24 h of rapamycin exposure was obtained in both U251MG and A172 cell lines, as reported in Supplementary Figure 6. Again, data from immune-fluorescence remarkably overlap with qRT-PCR data showing that the early neuronal marker βIII-tubulin steadily increases along a logarithmic scale of rapamycin doses, each dose being significantly more effective than the lower one. This is analytically evidenced in the graph of Figure 7 and it is representatively reported in pictures of Figures 7A-7P, where the increase in βIII-tubulin recruits the total amount of the cells, with each cell becoming more and more intensely stained along the dose-response curve (Figures 7D, 7H, 7L, 7P). The constant progression occurring dose-dependently for βIII-tubulin immune-fluorescence was replicated in both U251MG and A172 cells (Supplementary Figures 11 and 12, respectively). When βIII-tubulin immune-gold was carried out, the increase was confirmed showing detailed and fine distribution of this early neuronal marker. It is worth being noticed that this marker was already present, albeit at very low concentrations, in control cells (representative Figure 8A). This unexpected occurrence confirms what already reported in the literature [26, 27], thus toning down the absolute significance of this antigen as a fully reliable neuronal marker. In fact, βIII-tubulin may also occur quite ubiquitously in low amount owing a non specific significance. This is why we matched the βIII-tubulin data with NeuroD and NeuN since the simple βIII-tubulin immune-staining was already reported by Zhuang et al. [16]. Nonetheless, here rapamycin produces a strong and specific dose-dependent increase of βIII-tubulin staining, which augments significantly even for the highest dose of rapamycin (Figures 8B and 8C and graph of Figure 8). An increase in βIII-tubulin immune-gold was also observed in rapamycin-treated U251MG and A172 cells (Supplementary Figures 13 and 14, respectively), although in U251MG cell line this effect was significant only at the highest dose of rapamycin (1 μM). A similar trend was measured by semi-quantitative optical density of western blotting for βIII-tubulin in U87MG cells (Figure 9). It is worth to be mentioned that, among the various antigens we measured in these experiments, the neuronal antigens βIII-tubulin and NeuN do not reach a plateau with the highest dose of rapamycin. This is in striking contrast with the effects produced for the stemness antigen nestin which are almost complete at low doses of rapamycin. These data which differ slightly between cell lines, indicate that neuronal differentiation and the loss of stemness follow two separate dose-response curve, thus suggesting independent pathways, both under the regulation of mTOR, to be responsible for these phenomena. When the fine morphology of βIII-tubulin is analyzed, the protein was found in strong association with cytosolic filaments. As mentioned, to confirm the effects of rapamycin on the induction of early neuronal markers, we also counted the number of NeuroD positive cells under the effects of various doses of rapamycin, in both U87MG and A172 cell lines, as reported in Supplementary Figures 15 and 16, respectively. This marker is quite similar to βIII-tubulin concerning neuronal maturation [28–30]. The increase in NeuroD produced by rapamycin confirms what we measured for βIII-tubulin and lend substance to βIII-tubulin data obtained by Zhuang et al. [16].

### Rapamycin dose-dependently increases NeuN

**Figure 10:**
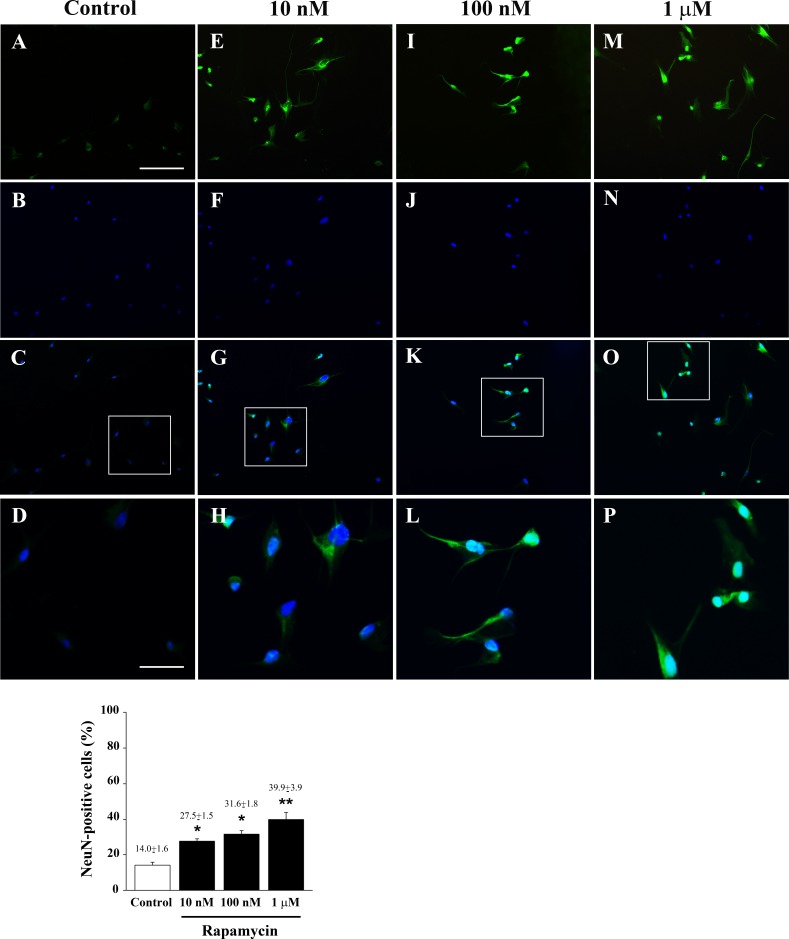
Rapamycin dose-dependently increases NeuN immune-fluorescence Immune-fluorescence of U87MG cells treated with vehicle **A**.-**D**. and rapamycin at the dose of 10 nM **E**.-**H**.; 100 nM **I**.-**L**.; 1 μM **M**.-**P**. In the first line cells were stained for the late post-mitotic neuronal marker NeuN. In the second line cells were stained for the nuclear dye DAPI. In the third line the merging between NeuN (green) and DAPI (blue) fluorescence is shown. In the fourth line a high magnification of the squared insert of line three is shown. The graph reports the dose-dependent rapamycin-induced increase in NeuN-positive cells. Values are given as the mean±S.E.M. Comparisons between groups were made by using one-way ANOVA with Scheffé post-hoc test. *P ≤ 0.05 *vs* control. **P ≤ 0.05 *vs* control and 10 nM rapamycin. Scale bars = (A-C, E-G, I-K, M-O) 155 μm; (D, H, L, P) 45 μm.

**Figure 11:**
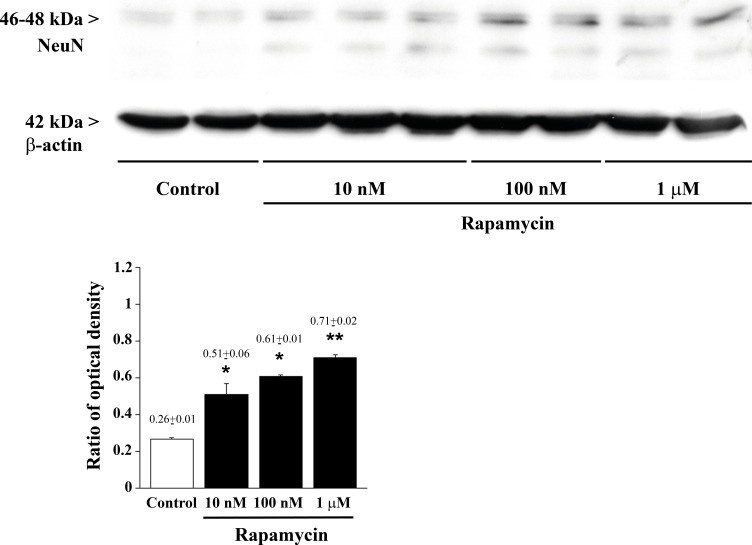
Rapamycin dose-dependently increases NeuN assessed by immune-blotting Representative immune-blots for the late neuronal marker NeuN and the housekeeping protein β-actin. The ratio between the optical densities of NeuN and β-actin is reported in the graph. Rapamycin increase NeuN dose-dependently. Values are given as the mean±S.E.M. Comparisons between groups were made by using one-way ANOVA with Bonferroni test. *P ≤ 0.05 *vs* control. **P ≤ 0.05 *vs* control and 10 nM rapamycin.

The induction of early neuronal markers staining neuronal precursors during a mitotic stage does no guarantee for the occurrence of a frank neurogenesis. Thus, to strengthen the early findings of Zhuang et al. [16] and our extended analysis of early neuronal markers presented so far, the post-mitotic antigen NeuN was extensively analyzed. In qRT-PCR experiments, the mRNA for the late (post-mitotic) neuronal marker NeuN [31–33] was quantified as steadily increasing dose-dependently under rapamycin exposure in U87MG cells (Figure 3) as well as in both U251MG and A172 cells at 24 h (Supplementary Figure 6). Remarkably, the transcript for NeuN possesses the highest sensitivity to mTOR inhibition among all other markers. In fact, the mRNA for this antigen was highly increased at 24 h and it was the only one to be modified at 4 h in U87MG cells, when the mRNA was unexpectedly decreased for the highest rapamycin doses. Immune-fluorescence for NeuN is shown in representative pictures from control and rapamycin-treated U87MG (Figure 10) and A172 cells (Supplementary Figure 17). Such an increase progresses up to the highest dose of rapamycin (Figure 10P and Supplementary Figure 17P), as counted in both graphs of Figure 10 and Supplementary Figure 17, which report a further net increase even for the highest dose of rapamycin. This trend was preserved in semi-quantitative counts reported for western blotting carried out in U87MG (Figure 11).

Western blotting for each specific antigen confirms the trend obtained with other methods. Nonetheless, when compared with quantitative procedures such as immune-electron microscopy, counts at immune-histochemistry, and qRT-PCR, data from western blotting are in line but with slight differences. This is not surprising due to variability of regression curves obtained by measuring optical density versus protein amount for each specific antigen. This is both antigen-dependent and it is inherently due to a “semi-quantitative procedure” which does not allow to draw a linear ratio (or any constant mathematical function) to correlate increasing optical density and increasing protein amount. In other words, protein measurement by western blotting is expressed by arbitrary units produced by optical density, which varies in the shape of regression curves for standard doses depending on each specific antigen/antibody complex and changes at various range of doses. These limits are inherent to the methods and need to take cautiously differences between groups which are measured for optical density (consistent increase or decrease rather than emphasizing slight variations). Strikingly, the data obtained through quantitative approaches we provided here are quite overlapping and they best define the slight effects, which, in any case, are similar to western blotting.

### Rapamycin does not modify GFAP

**Figure 12:**
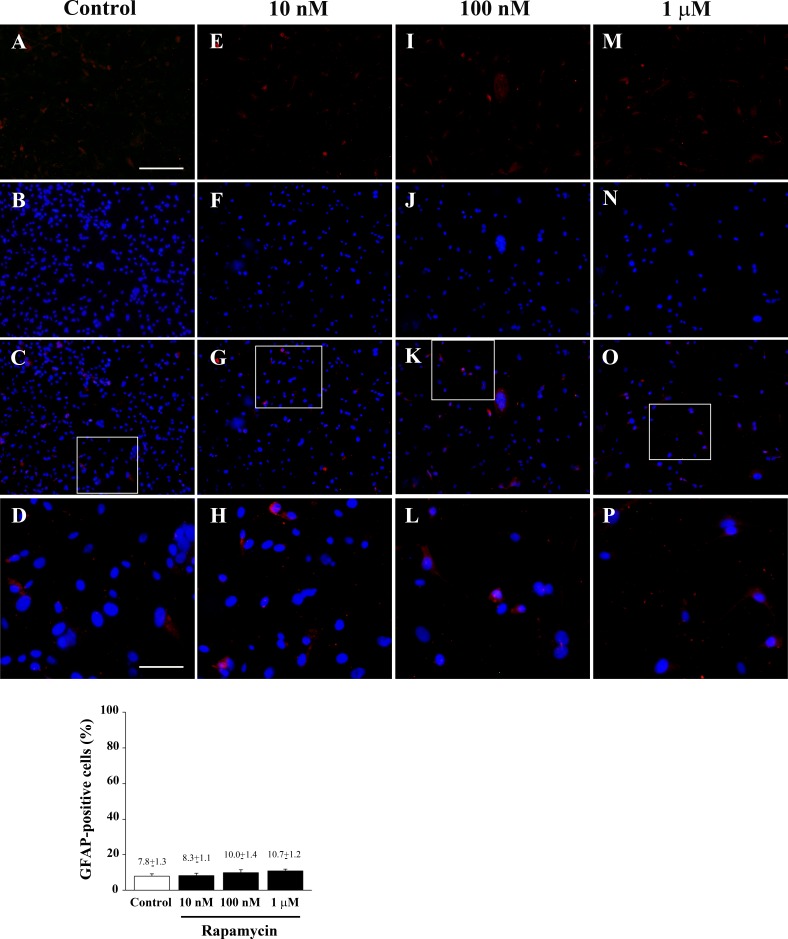
Rapamycin does not modify GFAP immune-fluorescence Immune-fluorescence of U87MG cells treated with vehicle **A**.-**D**. and rapamycin at the dose of 10 nM **E**.-**H**. 100 nM **I**.-**L**.; 1 μM **M**.-**P**. In the first line cells were stained for the glial marker GFAP. In the second line cells were stained for the nuclear dye DAPI. In the third line the merging between GFAP (red) and DAPI (blue) fluorescence is shown. In the fourth line a high magnification of the squared insert of line three shows that GFAP-positivity is not modified following treatment with rapamycin compared with baseline conditions. Counts reported in the graph confirm the lack of any effect. Values are given as the mean±S.E.M. Comparisons between groups were made by using one-way ANOVA with Scheffé post-hoc test. Scale bars = (A-C, E-G, I-K, M-O) 155 μm; (D, H, L, P) 45 μm.

**Figure 13:**
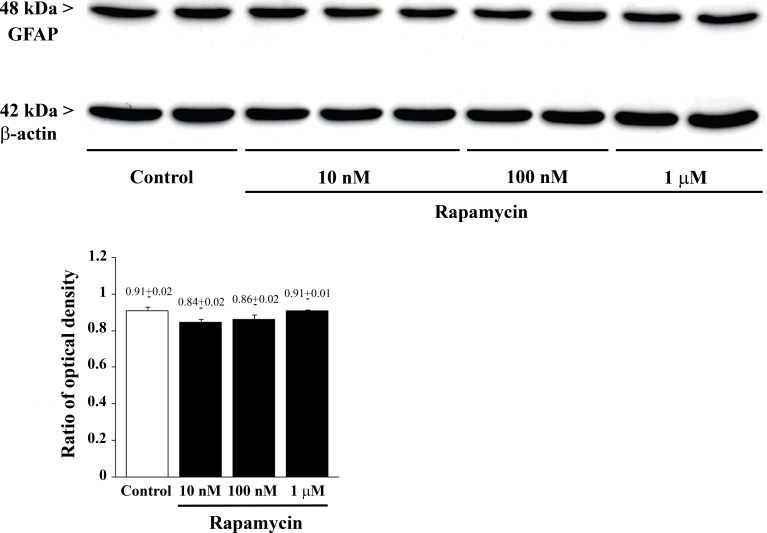
Rapamycin does not modify GFAP as assessed by immune-blotting Representative immune-blots of GFAP and the housekeeping protein β-actin in control and rapamycin-treated U87MG. The ratio between the optical densities of GFAP and β-actin is reported in the graph and it confirms that GFAP expression is not modified by rapamycin. Values are given as the mean±S.E.M. Comparisons between groups were made by using one-way ANOVA with Bonferroni test.

Increasing doses of rapamycin never alter the amount of GFAP positive cells which were barely detectable. Similarly, the GFAP signal which was clearly detected at western blotting was not modified by any dose of rapamycin (Figure 12 and Figure 13). These data indicate the lack of effects of rapamycin towards phenotype of glial activation, which is in line with previous data showing that mTOR inhibition does not induce glial differentiation [34–36].

### Effects of rapamycin on FBS-induced cell migration and pFAK expression

**Figure 14:**
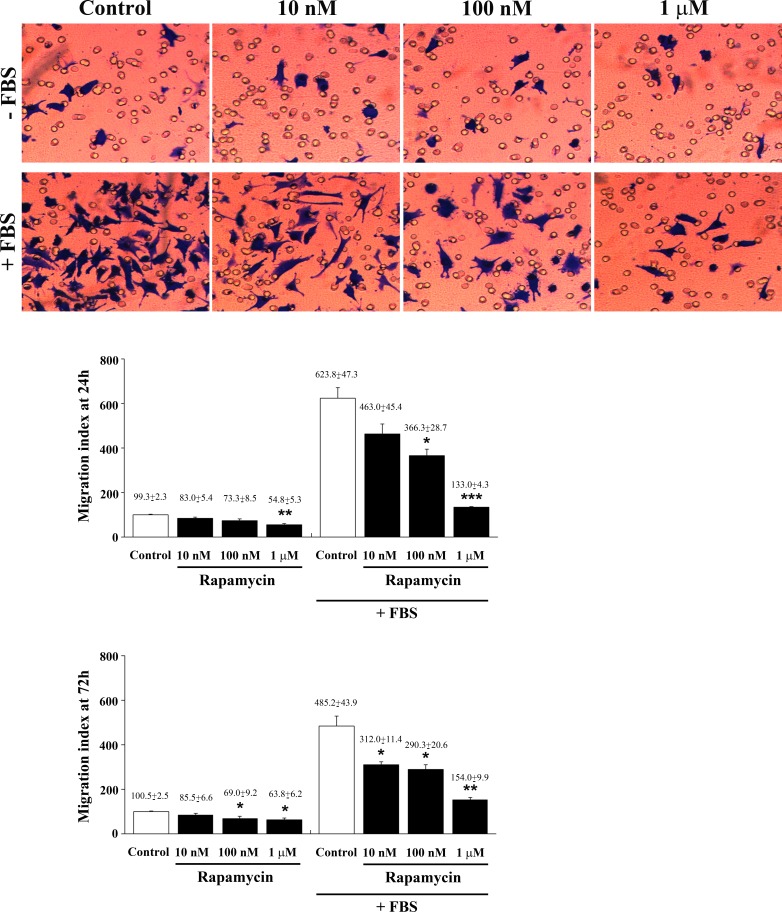
Rapamycin dose-dependently suppresses FBS-induced cell migration Representative images of migrated cells towards a medium without or with FBS for 3h. Graphs report the migration index of U87MG cells treated for 24 h or 72 h with different doses of rapamycin towards a medium without or with FBS for 3h. Data are expressed as percentage of migrated cells in comparison with control in the absence of FBS (sum of data obtained at 24 h and 72 h, taken as 100%). Data are the mean±S.E.M. Comparisons between groups were made by using one-way ANOVA. *P ≤ 0.05 *vs* respective control. **P ≤ 0.05 *v*s respective control and 10 nM rapamycin. ***P ≤ 0.05 *vs* respective control and rapamycin at 10 nM and 100 nM.

In seeking to provide a functional link between consistent formal evidence about rapamycin-induced cell differentiation and functional cell properties, we analyzed whether, in the same experimental conditions, fetal bovine serum (FBS)-induced migration of U87MG cells was similarly affected by different doses of rapamycin. In Figure 14 representative micrographs report a dose-dependent rapamycin-induced inhibition of FBS-induced migration which was calculated in the graph at 24 h and 72h of treatment. This was observed only for those cells migrating towards FBS, whereas it was not detectable in cells migrating towards a medium without FBS (with the exception of the highest doses). This suggests that rapamycin has specific effects on cell chemotaxis while is not specific for cell chemokinesis. Data related to cell migration obtained in U251MG and A172 cells at 24 h and 72 h after rapamycin exposure were reported in Supplementary Figures 18 and 19, respectively. Remarkably, in these additional cell lines a reduced motility was observed even within a medium without FBS for the lower doses of rapamycin. Since chemotaxis relies on the expression of the adhesion molecule pFAK, we carried out experiments on U87MG treated with different doses of rapamycin using pFAK immune-fluorescence. As shown in representative pictures of Supplementary Figure 20, in the presence of FBS the chemotaxis-related antigen was highly expressed, while such an expression was suppressed dose-dependently by rapamycin administration. This correlates with the suppression of cell migration measured in the graph of Figure 14. In detail, the highest doses of rapamycin produces an amount of pFAK expression comparable with control (non FBS exposed) cells (Supplementary Figure 20). Similar results were also obtained in U251MG cells (Supplementary Figure 21). In these sets of experiments, the lab carried out pilot studies to assess cell viability through the 3-(4,5-dimethylthiazol-2-yl)-2,5-diphenyltetrazolium bromide (MTT) assay in each cell line, as reported in Supplementary Figures 22 and 23. This was carried out to test the cell culture during migration experiments. In these experimental conditions at 24 h, and mostly at 72 h, a few cell death was measured according to MTT, which confirms a neglectable amount of cell death only for the highest rapamycin doses we previously measured using this assay. This is demonstrated to depend almost entirely on autophagy while apoptosis is neglectable [23].

### Rapamycin dose-dependently suppresses pS6

In order to validate the occurrence of mTOR inhibition under the effects of increasing doses of rapamycin, we performed an immune-blot analysis for the ribosomal protein p6S, commonly used as index of mTOR activity since it is located downstream to mTOR activation. Consistently with most of the dose-response curve produced by different methods reported in the previous paragraph, we found that rapamycin suppresses p6S protein expression already at the dose of 10 nM in each cell line (Supplementary Figures 24-26). Remarkably, in A172 cells we measured the most severe suppression which is consistent with the highest sensitivity of A172 cells to most of the effects induced by rapamycin. This strongly suggests that all these effects indeed depend on mTOR inhibition (Supplementary Figure 26).

## DISCUSSION

In the present study we report several lines of evidence, based on a number of different experimental procedures carried out in different GBM cell lines, which demonstrate the molecular effects produced by the powerful mTOR inhibitor, rapamycin. Rapamycin strongly modulates the expression of specific mRNA by stimulating the transcription of those genes related to neuronal differentiation while inhibiting the production of mRNA coding for the stemness antigen nestin. At the same time rapamycin produces a variety of phenotypic alterations, each one in a dose-dependent manner starting from very low doses of the compound. In fact, most of these effects were produced significantly by a dose of 10 nM which is normally produced by administering therapeutic doses of such mTOR inhibitor. Remarkably, when we analyzed the mTOR inhibition expressed by suppression of the downstream product of mTOR Complex 1 (mTORC1) activity p6S, the dose of 10 nM was fully effective. These results extend the effects of rapamycin on cell differentiation described by Zhuang et al. [16] who use only one very high dose rapamycin (200 nM) way higher compared with the therapeutic range. In fact, this is a very important point. According to the Stenton review, the therapeutic range recommended for rapamycin to produce its clinical effects is 12-20 mg/L [37]. When considering the molecular weight of rapamycin, 914.172, this corresponds to a therapeutic range expressed in moles between 13.13 and 21.88 nM. Remarkably, in the present study some effects we obtained following rapamycin in U87MG cell line reached a plateau already at the dose of 10 nM, which corresponds to a dose slightly below the therapeutic range (13-21 nM). When tested in A172 cell line the effects of rapamycin occurred even with lower doses, whereas when tested in another Uppsala derived cell line (U251MG) the effects produced by 10 nM were less pronounced but still highly significant. Thus, we provided evidence for a number of effects induced by rapamycin within its therapeutic range or even at slightly subthreshold doses.

The morphological evidence we provide here for the dose-dependent phenotypic shift represents the first demonstration that rapamycin, at a dosing plateau of 10 nM modifies GBM cell morphology. This is evident in U87MG cells, switching towards a neuronal like shape with a number of long branches arising from a pyramidal cell body gifted with a wide nucleus. Thus, phenotypic changes encompass rough variations in cell and nuclear size, cell shape and the amount and length of cell branching which occur when the cell line develops a neuron-like morphology. Changes in the shape of the cell body although occurring in other GBM cell lines were less pronounced when considering the number of cell branching. These neuron-like morphological alterations were paralleled by a dose-response loss of stemness as shown by rapamycin-induced suppression of stemness-related antigen nestin, which was quantified both as mRNA and protein detected as nestin immune-fluorescent and more carefully counted by stoichiometric quantitative assessment of nestin immune-gold electron microscopy. This was confirmed by semi-quantitative SDS-Page immune-blotting. Each experimental procedure provided similar results with the highest degree of coherency between quantitative methods (such as qRT-PCR and cell counts) compared with slight, non critical discrepancies detected by optical estimation of protein levels at western blots. Again, in most experimental setting the dose of 10 nM rapamycin was enough to produce the maximal nestin suppression. At immune-gold, only when rapamycin rose up to 1 μM we observed a further slight suppression of nestin. This second peak of effects is likely to escape from the canonical mTOR regulation. In fact, in A172 cells where the effects of rapamycin were mostly correlated to mTOR inhibition, the suppression of nestin immune-gold observed for 10 nM was identical to that obtained at 100 nM and 1 μM of rapamycin.

There is no doubt that most of the effects produced by rapamycin as an mTOR inhibitor in GBM cells are grounded on reverting a massive autophagy inhibition [21]. However, recent evidence demonstrates that other mechanisms, such as the amount of prion protein, may strongly affect the phenotype of GBM cells [38]. Interestingly, rapamycin is able to modulate prion protein metabolism [39], which provides an alternative pathway for rapamycin induced phenotypic shift of GBM cells.

This high sensitivity to phenotypic changes generated by rapamycin was paralleled by the expression of the cell adhesion antigen pFAK. This suggests similar biochemical cascades regulating both antigens which suggests a connection between differentiation and cell migration. Some phenotypic changes occur with a wider dose-response curve. Both βIII-tubulin as well as NeuroD can be considered as early neuronal markers according to current literature. The significance of these markers is quite similar for neuronal maturation being typical of ongoing neurogenesis still at mitotic stages, being NeuroD more restricted to some neuronal populations than βIII-tubulin [28–30]. Both markers, despite increasing robustly following the lowest dose of rapamycin, continued to increase significantly up to higher doses of the mTOR inhibitor. The neuronal antigen NeuN, which is considered as a late neuronal marker, specifically labelling post-mitotic neurons [31–33], possesses a similar progressive increase. The increase in NeuN is even more pronounced compared with early neuronal markers for the highest doses of rapamycin and it does not provide a clear evidence for the occurrence of a plateau even for the highest dose of rapamycin (1 μM) we used in the present study, which exceeds the sole dose of 200 nM which was used by Zhuang et al. [16]. Altogether our data suggest that the loss of stemness and the different steps in neuronal maturation are all regulated by mTOR. However, marked difference exist in the steepness between the dose-response curves of these phenomena, which suggests additional biochemical cascades to contribute differently in each effect. Altogether these data produced by therapeutic doses of rapamycin on GBM cells shown here provide a useful insight to plan combined therapy for the treatment of GBM by using rapamycin or other rapalogs, which are currently ongoing, mostly based on empiricism. The effects of mTOR regulation do not seem to involve the expression of GFAP, which confirms what previously reported. It should be taken into account that, in mature neuronal circuitries the inhibition of mTOR seems to produce a suppressing effect on GFAP [34–36]. In this biological matrix we could not confirm an inhibitory action of mTOR inhibition on GFAP expression. It is likely that there is no regulation of this gene in this cell type (as confirmed by the absence of mRNA alterations). Thus, although it might surprise that rapamycin (known as a suppressor of glia proliferation) does not modify GFAP levels, it is likely that the occurrence of small amount of GFAP in control cells is not the result of a canonical regulation of gene expression but it rather occurs due to the aberrancy of these cells.

The present study provides strong consistency in experimental evidence which encompasses a variety of methods used to assess various effects produced by rapamycin. In fact, all quantitative measurements were remarkably similar with slight variations when expressing data in arbitrary units. In fact, optical density used for quantify western blotting carries the intrinsic bias of non-linear distribution of optical density related to different protein amount. This is witnessed by the lack of a specific mathematical equation for the regression curves drawn for protein amount and optical density along a dose-response curve. Anyhow, despite these slight discrepancies, the statistical significance was consistent with data produced by quantitative approaches.

Such an extensive experimental collection of phenotypic changes was mandatory in order to move up to *in vivo* system to validate the outcomes of each specific finding. Remarkably, we replicated all the results in different GBM cell lines. At this point one might consider which experimental finding deserves at most to be translated *in vivo*. The experiment we feel the most important to be carried out *in vivo* consists in measuring in experimental tumors the neuronal differentiation under the effects of rapamycin. Indeed, we just published on line preliminary evidence showing that rapamycin reduces tumor growth and may revert tumor volume by promoting the integration of a glioblastoma xenograft into normal nervous tissue [40]. This specific issue deserves further in depth investigation.

All phenotypic changes we report in the present manuscript are expected to lead to significant outcomes when challenged in a model of tumor infiltration. For instance, chemotaxis which is specifically inhibited by very low doses of rapamycin along with inhibition in the expression of the cell adhesion molecule pFAK is supposed to alter tumor infiltration.

A number of studies demonstrate that mTOR inhibition increases radio- and chemo-sensitivity [16, 17, 41, 42] of GBM cells. For instance, Zhuang et al. [16] demonstrated that rapamycin may reduce the resistance to radiotherapy through an mTOR-dependent mechanism, which involves up-regulation of the autophagy pathway, which in turn would be responsible for the expression of markers for neuronal differentiation.

A number of trials are in progress to evaluate the effects of rapamycin analogs (rapalogs) and other phosphoinositide-3-kinase (PI3K)/mTOR pathway inhibitors as combined therapy to improve the outcome of chemo- and radio-therapy [11, 36, 43–45]. In fact, when considering all the properties of stem-like GBM-initiating cells such as cell proliferation, expression of stemness antigens as well as loss of cell aggregation and increased cell migration, all of them are affected by mTOR inhibition [5, 6, 9, 16, 23, 24].

However, the effects of these drugs may be lost due to development of resistance against mTOR inhibitors which depends on the overexpression of cyclin [46–48]. In this case it is demonstrated that sensitivity to mTOR inhibitors can be reproduced by administering inhibitors of internal ribosome entry site (IRES)-dependent which impede cyclin D1 and c-MYC translation [47, 49]. The significance of the present study is directly related to get an increased awareness of basic mechanisms which relate mTOR activity and the biology of GBM cells. Nonetheless, the findings we obtained here possess an inherent significance which applies to a variety of cells, mainly neurons under the effects of mTOR regulation. In fact, the ability to promote neuronal differentiation might be viewed as a novel therapeutic pathway to approach neuronal regeneration. This is suggested by the different steps promoted by rapamycin which passes through stemness up to early and late post mitotic neurogenesis. In this way our data may be regarded also as a novel path to rejuvenate the brain which might be useful in aging disorders.

### Additional mechanisms for mTOR modulation on neuronal regeneration

Hyperactivation of the mTOR pathway is considered a common feature in several tumors and represents the major molecular alteration in glioblastoma cells. However, recent findings indicate that cell growth and proliferation induced by mTOR might be linked to neuronal regeneration.

In particular, mTOR regulates axonal regeneration of retinal ganglion cells following optic nerve crush [50, 51] and enhances regeneration of the corticospinal tract [52–55]. Some of these effects are obtained by deleting the phosphatase and tensin homolog PTEN, the tumor suppressor gene which acts upstream to mTOR in the mTOR pathway.

In fact inhibition of mTOR is critical for neuronal differentiation.

In a transgenic model of tuberous sclerosis, characterized by hyperactivation of mTORC1, early application of rapamycin during neuroectodermal differentiation is able to correct aberrant neuronal differentiation [56]. Similarly, in a mouse model of Angelman syndrome, characterized by high levels of mTORC1 activity, rapamycin normalizes the synaptic signaling, regulates synaptic-related cytoskeletal elements and improves dendritic spine morphology of Purkinje cells, and improves motor performance [57].

## MATERIALS AND METHODS

### Cell cultures

The glioblastoma cell lines U87MG were obtained from Cell Bank (IRCC San Martino-IST, Genova). The cells were grown in the growth medium DMEM (Sigma Aldrich, Milan, Italy) supplemented with 10% Fetal bovine serum (FBS), 1% of MEM Non-Essential Amino-Acid (MEM-NEAA), penicillin (50 IU/mL) and 100 μg streptomycin (Sigma). Cells were maintained at 37°C in a humidified atmosphere containing 5% CO_2_ and the medium was renewed 2 times per week.

The glioblastoma cell lines U251MG were obtained from the European Collection of Authenticated Cell Cultures (ECACC) and maintained in EMEM, 2mM L-Glutamine, 1% of MEM-NEAA, 1mM Sodium Pyruvate (NaP), 10% FBS, 100 IU/ml penicillin and 100 mg streptomycin, at 37°C, 5% of CO_2_ and 95% of humidity.

The glioblastoma cell lines A172 were obtained from the European Collection of Authenticated Cell Cultures (ECACC) and from Cell Bank (IRCC San Martino-IST, Genova) and maintained in Modified Eagle's Medium (Euroclone, Milan, Italy) supplemented with 10% FBS, 2 mM L-glutamine, 100 IU/ml penicillin and 100 mg streptomycin, at 37°C, 5% of CO_2_ and 95% of humidity.

For cell count and immune-fluorescence experiments 5×10^4^ U87MG, U251MG and A172 cells were seeded on cover slips which were put down within 24-well plates in a final volume of 1 ml/well.

For electron microscopy, qRT-PCR and western blots assay, 1×10^6^ cells from each cell line were seeded in 6-well plates in a final volume of 2 ml/well.

### Rapamycin treatments

Twenty-four h after seeding, cells were treated with increasing doses of rapamycin (namely 1 nM, 10 nM, 100 nM and 1 μM) for 24 h. Dilutions of rapamycin were obtained by a stock solution (1 mM of rapamycin dissolved in culture medium containing 10% dimethyl sulfoxide, DMSO). Control cells were maintained in culture medium containing 0.01% DMSO.

### Light microscopy and immune-cytochemistry

At the end of the treatments, culture medium was washed out. For each treatment, some cells were left in the same well plate, rinsed in PBS and rapidly observed under light microscope without staining, in order to document the status of unfixed cultures.

The remaining cells were fixed with 4% paraformaldehyde in PBS for 15 min and then stained with H&E or immune-fluorescence.

Haematoxylin & Eosin. After fixation cells were plunged in the Haematoxylin solution (Sigma) for 20 min, washed in running water, and then immersed in the Eosin solution (Sigma) for a few minutes. Finally, cells were dehydrated in increasing alcohol solutions, clarified in xylene and covered with DPX mounting medium (Sigma).

Immune-fluorescence for stem-like and differentiation antigens. The following primary antibodies (Ab-I) were used: rabbit polyclonal anti-nestin Ab-I (Abcam, Cambridge, UK), for undifferentiated, stem-like cells [58]; mouse monoclonal anti-glial fibrillary acidic protein (GFAP) Ab-I (Sigma), as a marker of glial cells; mouse monoclonal anti-βIII-tubulin Ab-I (Millipore, Billerica, MA, USA) and anti-NeuroD Ab-I (Millipore) as early (immature) neuronal markers (Bédard and Parent, 2004); mouse monoclonal anti-NeuN Ab-I (Millipore) as late neuronal marker, which specifically label post-mitotic neurons [31–33]. Cells were first permeabilized by Triton X 0.1% for 15 min in PBS and then incubated in a blocking solution containing 10% normal goat serum (NGS) in PBS for 1 h at room temperature (RT). Cells were successively incubated with the Ab-I solution overnight at 4°C. The different Ab-I solutions were prepared in PBS containing 2% NGS. The Ab-I were diluted as it follows: anti-nestin Ab-I (1:200), anti-GFAP Ab-I (1:400), anti-βIII-tubulin Ab-I (1:200), anti-NeuN Ab-I (1:50), and anti-NeuroD (1:100). After washing out with PBS, the reaction with the Ab-I were revealed by using the anti-mouse or anti-rabbit fluorescent secondary antibody Alexa Fluor 488 (1:200, Life Technologies, Carlsbad, CA, USA) and the anti-mouse fluorescent secondary antibody Alexa Fluor 546 (1:200, Life Technologies) for 1 h and 30 min. To visualize the cell nuclei, cells were finally incubated for 5 min with the nuclear dye DAPI (Sigma) diluted 1:1000 in distillated water. All these reactions were carried out within the well plate. After washing in PBS, slices were gently pulled out and transferred on a coverslip and mounted with the mounting medium Fluoroshield (Sigma).

Slices were observed using the Nikon Eclipse 80i light microscope equipped with a fluorescent lamp and a digital camera connected to the NIS Elements software for image analysis (Nikon, Tokyo, Japan).

DAPI- and nestin-stained pictures were acquired independently and then they were merged using GIMP (GNU Image Manipulation Program) or NIS Elements software (Nikon). Final image panels were prepared using Photoshop.

Immune-fluorescence for the adhaesion protein phospho-FAK (pFAK). Cells (5×10^4^) were plated on coverslips and treated with different rapamycin concentration in presence of 10% FBS; after 24 h cells were washed in PBS, fixed in 4% PFA, permeabilized (Triton X-100 0.1%) and blocked (1% BSA) for 1 h at RT, and incubated overnight at 4°C with pFAK (Tyr397, Santa Cruz Biotechnology, USA) 1:500. Cells were stained with the fluorophore-conjugated secondary antibody and Hoechst for nuclei visualization and analyzed using fluorescence microscopy. pFAK levels were semi-quantitatively analyzed measuring the mean fluorescence intensity (MFI) of each signal with a 60x magnification.

### Cell counts and morphometry at light microscopy

In order to evaluate cell number, 5×10^3^ cells were seeded and treated with increasing doses of rapamycin. At the end of the treatments, 25 μl of cell suspension were added to 37.5 μl PBS containing 1% of trypan blue (62.5 μl). Cells were incubated for 5 min at room temperature, and 10 μl of this solution were used to perform cell count using a Bürker glass chamber. Values were expressed as number of cells/ml.

The number and length of cell branching was measured in H&E-stained cells and they were related to a total of 50 cells/group at 40 x magnification for each cell line. The length of cell branching was measured using the free software IMAGEJ.

Counts of immune-fluorescent cells were carried out at light microscopy at 20 x magnification. Values were presented as the mean percentage of immune-positive cells which were counted within 5 distinct microscopic fields, where cell distribution appeared without overlapping, allowing to distinctly visualize all cells. This allowed us to count over 500 cells for each experimental group.

Measures were carried out by two observers, unaware of the treatments. All data were obtained in triplicate.

### Transmission Electron Microscopy

U87MG cells were centrifuged at 1,000 g for 5 min. After removal of the supernatant, pellet was thoroughly rinsed in PBS. Fixation was carried out with a solution containing 2.0% paraformaldehyde/0.1% glutaraldehyde in 0.1M PBS (pH 7.4) for 90 min at 4°C. This fixing solution allows a minimal cover of antigen epitopes preserving the morphology of the tissue [59]. Specimens were post-fixed in 1% OsO_4_ for 1 h at 4°C; they were dehydrated in ethanol and embedded in Epoxy-resin.

For ultrastructural morphometry, sections were examined directly at transmission electron microscopy (TEM) at a magnification of 8,000x. Each grid contained non-serial sections to count at least ten cells.

Several grids were observed in order to obtain a total number of at least 50 cells for each experimental group.

### Post-embedding procedure

Specimens were post-fixed in 1% OsO_4_ for 1h at 4°C and then they were dehydrated in ethanol and embedded in epoxy resin. Ultrathin sections were cut at ultramicrotome. Similarly to fixing, post-fixing and embedding procedures were validated on pilot studies and they were reported by current literature as optimal conditions for immune-gold-based ultrastructural morphometry. The method employed here is a “non-classical” method, which combines aldehyde and mild OsO_4_ as first and second fixing steps allows minimal epitope covering while preserving cell architecture and providing an optimal “contrast effect” of various cell compartments. This method allows preserving sub-cellular structures with acceptable epitope integrity [60, 61].

Osmium enhances the contrast of various cytosolic compartments by delineating phospholipid membranes, and it prevents the formation of membranous artifacts, which may mimic autophagy vacuoles [62].

Post-fixed samples were then embedded using epoxy resin, since it is well-established and commonly used as embedding media for TEM, allowing an optimal ultrastructural resolution.

The post-embedding was carried out collecting ultrathin sections on nickel grids and incubating them in aqueous saturated sodium metaperiodate (NaIO_4_) at room temperature. NaIO_4_ is an oxidizing agent, which attacks the hydrophobic alkane side-chains of epoxy resin making the sections more hydrophilic and allowing a closer contact between immune-gold-conjugated antibodies and the antigens exposed on the surface of each section [60]. This step abets the detection of specific immune-gold placement within a context of subcellular integrity, which allows counting molecules within well-delineated specific cell compartments.

### Immune-cytochemistry at electron microscopy

Grids were incubated with cold PBS containing 10% goat serum and 0.2% saponin to block non-specific antigenic sites for 20 min at room temperature.

Following a blocking step, samples were incubated with Ab-I: anti-βIII-tubulin (Millipore, diluited 1:50); anti-nestin (Abcam, diluited 1:50). Incubations were carried out in ice cold PBS containing 1% goat serum and 0.2% saponin in a humidified chamber overnight at 4°C.

Ultrathin sections were incubated with gold-conjugated secondary antibodies (10 nm and 20 nm gold particles, Sigma) diluted 1:20 in PBS containing 1% goat serum and 0.2% saponin for 1 hour, at room temperature.

After rinsing in PBS, grids were incubated with 1% glutaraldehyde for 3 min, they were washed in distilled water (to remove trace of salts and preventing precipitation of uranyl acetate), and they contrasted with uranyl acetate (saturated solution in distilled water) and lead citrate to be finally observed by using a Jeol JEM SX 100 electron microscope (Jeol). Control sections were obtained by omitting the Ab-I and by incubating with the secondary antibody only.

### Count of immune-gold particles

Pellets from cell lines allow an appropriate condition for quantitative morphometry since cells are randomly oriented on ultrathin sections owing a similar chance to be selected. In fact, counts obtained from different cell pellets possess a very small standard error [59, 63].

Finally, specimens consist of pellet blocks, some of which were randomly selected for sectioning, and some of the ultrathin sections were collected on grids for microscopy examination.

Count of immune-gold particles placed in the cytosol and in the nucleus, was carried out by electron microscopy at 8,000X magnification according to Lucocq et al. [64]. This magnification corresponds to the minimal magnification which allows identification of gold particles and cell compartments.

Each grid contained non-serial sections, and for each grid we observed an average of 10 cells. Several grids were observed in order to obtain a total number of 50 cells from each experimental group.

For quantifying immune-gold in ultrathin sections each observer started from the corner of a squared grid, to scan through equally spaced parallel sweeps across the specimens made up of a whole sectioned pellet. Random selection of the scanning erase on average those bias due to variations of cell density or intensity of gold labeling. For βIII-tubulin or nestin, in each cell the number of anti-βIII-tubulin or anti-nestin immune-gold particles placed within cytoplasm and nucleus was counted, and it was expressed by the mean value of immune-gold particles for cell out of 50 cell counted.

### RNA extraction for qRT-PCR

Total RNA was isolated from cultured cells using TRIzol Reagent (Invitrogen, Life Technologies) according to the manufacturer's instructions. The concentration and purity of RNA samples were determined using Nanodrop 2000 (Thermo Scientific, Life Technologies). Total RNA (1 μg) was reverse transcribed (RT) with SuperScript® VILO™ (Invitrogen, Life Technologies) with Oligo dT primers.

### qReal-Time-PCR

Amplifcation and detection were performed on a CFX Connect^TM^ Real Time System (Bio-Rad, Hercules, CA, USA). PCR mix including 10 μl SYBR Green PCR Master (Applied Biosystems, Foster City, CA, USA), 0,5 μM of each primer and 0,8 μl of RT reaction mix, was amplified as follows: 50°C for 1 minute, 95°C for 10 min followed by 40 cycles of 95°C for 30 s, 54°C for 1 m. The following primers have been designed using GenBank (http://www.ncbi.nlm.nih.gov/):

Nestin (NM_006617)5′-TGGAGCAGGAGAAACAGG-3′;5′-ATCTCTGCATCTACAGCAG-3′,TUBB3 (NM_006086) 5′-AGTCATCAGTGATGAGCAT-3′;5′-CAGGCCTGAAGAGATGTC-3′, NeuN (NM_001082575) 5′-GCGGCTACACGTCTCCAACATC-3′;5′-ATCGTCCCATTCAGCTTCTCCC-3′, B –Actin (NM_001101.3) 5′-GTGCGTGACATTAAGGAG-3′;5′-GCCAGACAGCACTGTGT-3′, B-Globin (NM_000518.4) 5′-CTAAGGTGAAGGCTCATG-3′;5′-GATAGGCAGCCTGCACT-3′.

Positive controls (DNA), negative control (distilled water), and RT-negative controls (total RNA sample) were included in each run.

The relative quantification was calculated using comparative Ct method (also known as the ΔΔCT method) [65, 66], and beta-globin and beta-actin were selected as internal references. Ct values correspond to mean values of each PCR performed in triplicate. Gene expression was confirmed in two independent experiments using both beta-globin and beta-actin as internal references.

### SDS-PAGE western blotting

Cells were homogenized at 4°C in ice-cold lysis buffer with phosphatase and protease inhibitor. One microliter of homogenates was used for protein determinations. Proteins (30 μg) were separated on sodium dodecyl sulphate polyacrylamide gels (7.5%) and transferred on Immuno-PVDF membranes (Bio-Rad, Milan, Italy) for 1 h. Filters were blocked overnight in Tween-20 Tris-buffered saline (TTBS) (100 mM Tris-HCl, 0.9% NaCl, 1% Tween 20, pH 7.4) containing 5% non-fat dry milk. Blots were incubated overnight at 4°C with primary antibody mouse anti-GFAP (1:300, Sigma), mouse anti-β-III-tubulin (1:800, Millipore), rabbit anti-nestin (1:2500, Millipore), rabbit anti-NeuN (1:2000, Millipore) or rabbit anti-pS6 (1:2000, Cell Signaling). For the normalization we have used β-actin. Blots were incubated with primary mouse monoclonal anti β-actin antibody (1:50000, Sigma) for 1 h at room temperature. Filter was washed 3 times with TTBS buffer and then incubated for 1 h with secondary peroxidase-coupled antibodies (anti-mouse, 1:7000; Calbiochem, Milan, Italy). Immunostaining was revealed by enhanced chemiluminescence luminosity (GE Healthcare, Milan, Italy). Densitometric analysis was performed with ImageJ software. Data were obtained in duplicate or triplicate.

### Chemotaxis assay

In parallel cultures from different laboratories 2.5×10^5^ cells were seeded on 35-mm Petri dishes in growth medium specific for each cell line. After 24 h, half of medium was replaced with fresh medium containing different treatments: vehicle (DMSO) or rapamycin at the final concentration of 10 nM, 100 nM or 1 μM; during longer treatments DMSO or rapamycin were added every 24 h.

Chemotaxis experiments were performed with treated cells after 24 h and 72 h of treatment. Briefly, cells were trypsinized and re-suspended in chemotaxis medium (DMEM with 100 U/ml penicillin, 0.1 mg/ml streptomycin, 0.1% BSA and 25mM Hepes, pH 7.4) at a density of 2×10^5^/ml. Cells were then plated on an 8μm pore polycarbonate membrane, in presence of chemotaxis medium with or without 10% FBS in the bottom well of a Boyden chamber.

After 3h of incubation at 37°C, membranes were washed in PBS, treated with ice-cold 10% trichloroacetic acid for 1 min and cells adhering to the upper side of the filter were scraped off, whereas cells on the lower side were stained with a solution containing 50% isopropanol, 1% formic acid, and 0.5% (wt/vol) brilliant blue R 250. Stained cells were counted with a 20x objective.

Results are expressed as percentage of migrated cells in comparison with control (taken as 100%) and each count derives from the mean±SEM of 4-5 independent experiments.

### MTT assay

In the experimental conditions used to test cell migration viability was measured in rapamycin-treated cells with MTT assay a 24 h and 72 h as reported in Supplementary Figures 22 and 23. Briefly, 5 × 10^4^ cells were seeded in 24-well plate and, after 24h, half of medium was replaced with fresh medium containing different treatments: vehicle (DMSO) or rapamycin at the final concentration of 10 nM, 100 nM or 1 μM; during longer treatments DMSO or rapamycin were added every 24 h.

At the end of treatments, medium was changed with fresh medium containing 0.5 mg/ml of MTT and incubated at 37°C for 2 h; medium was then aspired and cells were treated with DMSO for 10 min.

Samples absorbance was analyzed in a microplate reader at 570 and 630 nm to subtract background. Results are expressed as Viability Index, taking as 100% untreated cells in control condition.

### Statistical analysis

Statistical analysis was performed using a statistical software (GraphPad Prism Software, version 5.0, apart from qRT-PCR in which the lab used the version 6.0).

Measures related to cell number and immunopositivity for stem-like and differentiation antigens were expressed as the mean percentage ± S.E.M. For cell morphometry, values were expressed as the mean number ± S.E.M. and the mean length ± S.E.M.

For ultrastructural morphometry related to the count of βIII-tubulin and nestin immune-gold particles, values were expressed as absolute numbers. Data are reported as the mean or the mean percentage ± S.E.M. Inferential statistics to compare groups was carried out by using One-way analysis of variance, ANOVA, with Sheffè's post-hoc analysis. Differences between groups were considered statistically significant when the null hypothesis (H_0_) was P ≤ 0.05.

For qRT-PCR experiments statistical analyses using one-way analysis of variance (ANOVA) followed by Bonferroni test using GraphPad Prism version 6.0 has been used to test differences in gene expression in basal and treated cells (P<0.05).

For western blot experiments, values of optical density were presented as the mean ± S.E.M. Analysis of variance (one-way ANOVA) was used to compare the experimental conditions and Bonferroni test was used to compare the mean values for all groups. The null hypothesis was rejected for P ≤ 0.05.

Data on cell migration were statistically analysed using One-Way ANOVA (N= 4 for chemotaxis experiments, N=3 for pFAK immune-fluorescence).

## SUPPLEMENTARY MATERIALS FIGURES AND TABLES



## Abbreviations

Ab-I: primary antibody
DMEM: Dulbecco's modified minimal essential medium
DMSO: dimethyl sulfoxide
ECACC: European Collection of Authenticated Cell Cultures
EMEM: Eagle's minimal essential medium
FBS: fetal bovin serum
pFAK: phospho-FAK
GBM: glioblastoma multiforme
GFAP: glial fibrillary acidic protein
GIMP: GNU Image Manipulation Program
H&E: Haematoxylin and Eosin
IRES: internal ribosome entry site
MEM: minimal essential medium
MGMT: O^6^-methylguanine-DNA-methyltransferase
MFI: mean fluorescence intensity
MTT: 3-(4,5-dimethylthiazol-2-yl)-2,5-diphenyltetrazolium bromide
NEAA: Non-Essential Amino-Acid
NIS: Nikon Imaging Software
PI3K: phosphoinositide-3-kinase
PTEN: phosphatase and tensin homolog
qRT-PCR: quantitative real time-polymerase chain reaction
TEM: transmission electron microscopy
mTOR: mammalian Target Of Rapamycin
mTORC1: mammalian Target Of Rapamycin Complex 1
