# Supplementary material for: Rapamycin promotes differentiation increasing βIII-tubulin, NeuN, and NeuroD while suppressing nestin expression in glioblastoma cells
Source: Oncotarget. 2017 Mar 18;8(18):29574–99. doi: 10.18632/oncotarget.15906 (PMC5444688; doi:10.18632/oncotarget.15906)
Supplement: Supplementary file 1 [file oncotarget-08-29574-s001.pdf]

Rapamycin promotes differentiation increasing βIII-tubulin, NeuN, and NeuroD while suppressing nestin expression in glioblastoma cells

Supplementary Material

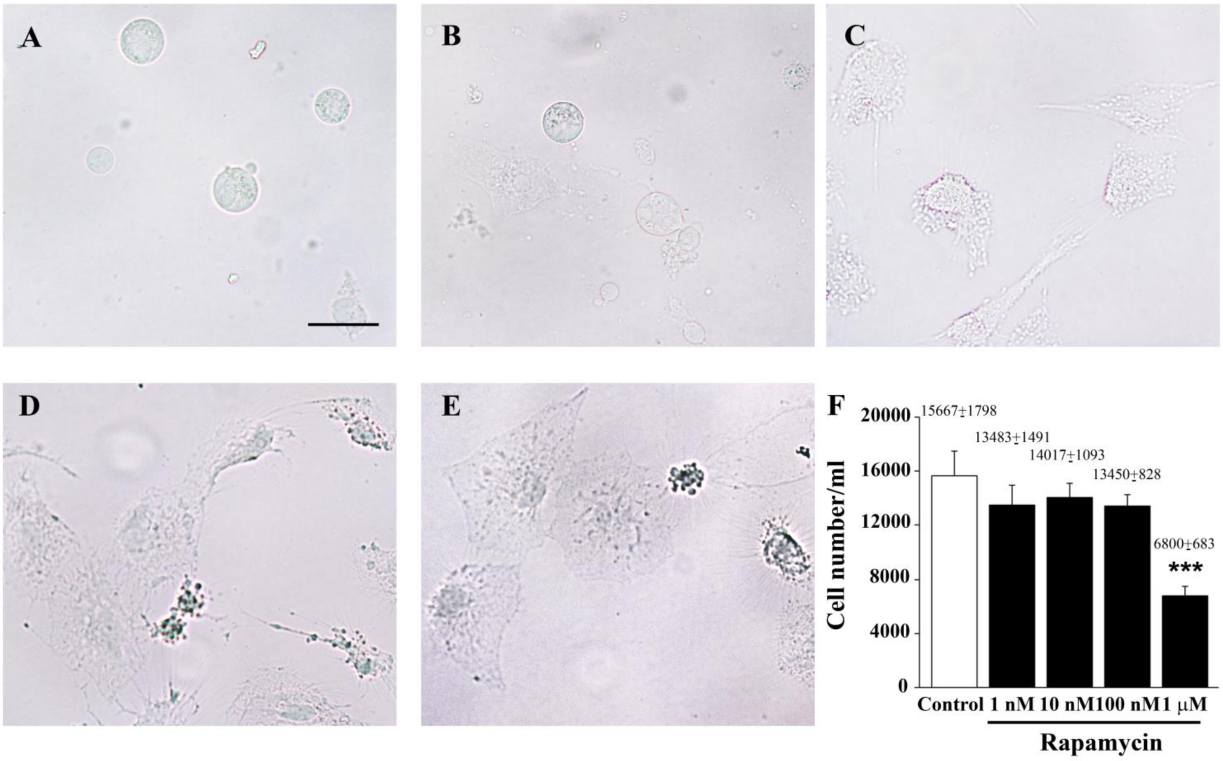

Supplementary Figure 1. Rapamycin dose-dependently reduces U251MG cell number.

Representative pictures of non-fixed/non-stained U251MG cells treated with vehicle (control) (**A**) and following 24 h of treatment with various doses of rapamycin: 1 nM (**B**), 10 nM (**C**), 100 nM (**D**), 1 µM (**E**). The graph reports the total number of cells which were counted in 1 ml, by using the Bürker chamber (**F**).

Values are given as the mean±S.E.M. Comparisons between groups were made by using one-way ANOVA with Scheffé post-hoc test.

***P≤0.05 *vs* control and rapamycin at 1 nM and 10 nM.

Scale bar = 35 µm.

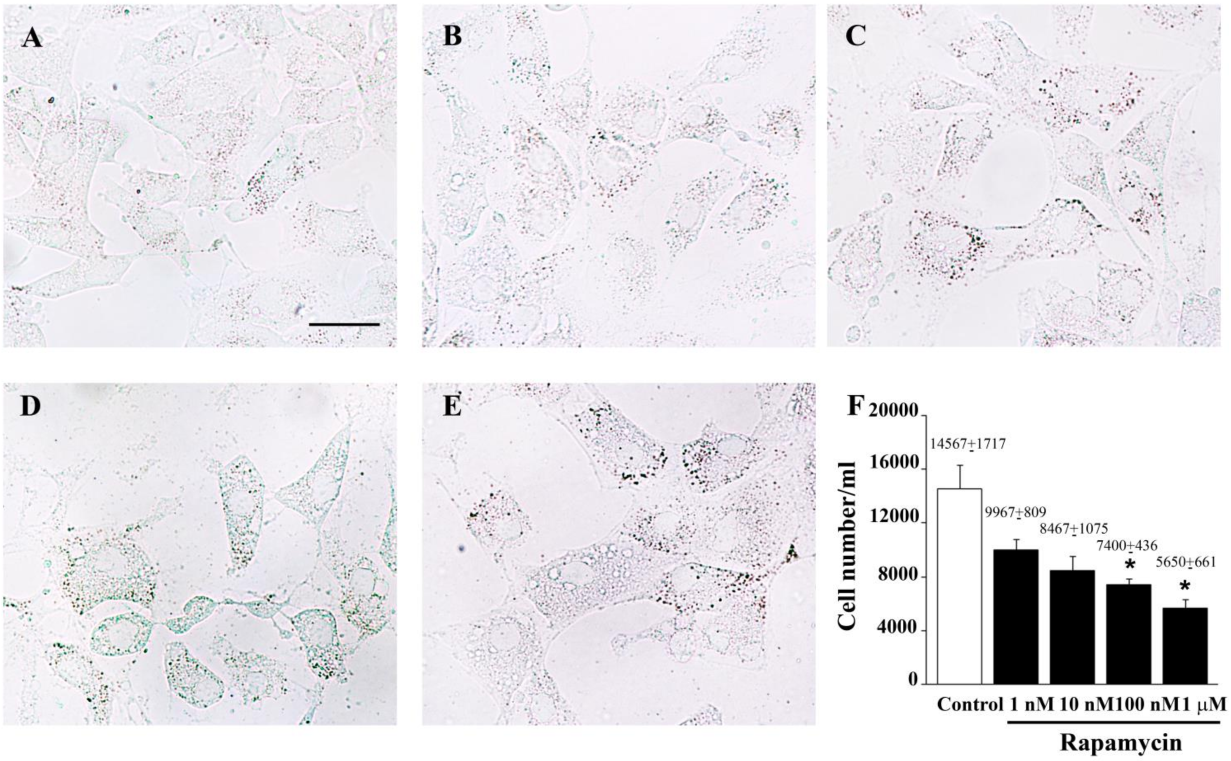

Supplementary Figure 2. Rapamycin dose-dependently reduces A172 cell number.

Representative pictures of non-fixed/non-stained A172 cells treated with vehicle (control) (**A**) and following 24 h of treatment with various doses of rapamycin: 1 nM (**B**), 10 nM (**C**), 100 nM (**D**), 1 μM (**E**). The graph reports the total number of cells which were counted in 1 ml, by using the Bürker chamber (**F**).

Values are given as the mean±S.E.M. Comparisons between groups were made by using one-way ANOVA with Scheffé post-hoc test.

*$P \leq 0.05$ *vs* control.

Scale bar = 35 μm.

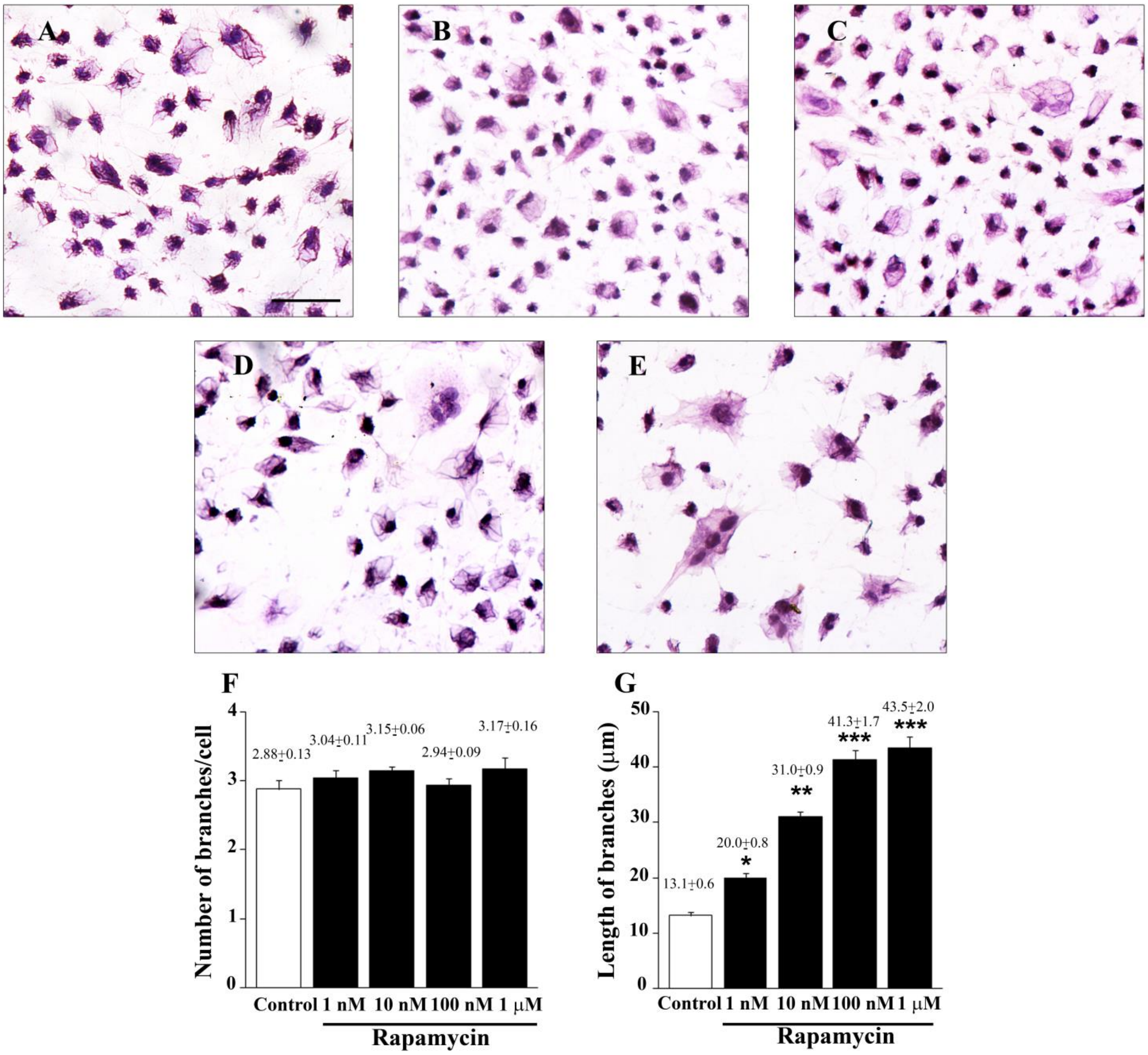

Supplementary Figure 3. Rapamycin dose-dependently increases the length of U251MG cell branching.

Representative pictures of H&E-stained U251MG cells. (**A**) Control cells show a well evident cytoskeleton forming an intensely basophilic network within a very pale cytosol. A number of thin branches develop from the cell body, which generally shows a very irregular shape. Rapamycin treatment dose-dependently increases the size of cell body which is associated with an increase in the length of cell branching but does not affect the number of cell branches, 1 nM (**B**), 10 nM (**C**), 100 nM (**D**) and 1 μM (**E**) of rapamycin. The graphs report the number of cell branches per cell (**F**), which is not affected by rapamycin, and the length of cell branches (**G**).

Values are given as the mean±S.E.M. Comparisons between groups are made by using one-way ANOVA with Scheffé post-hoc test.

*P≤0.05 *vs* control.

**P≤0.05 *vs* control and 1nM rapamycin.

***P≤0.05 *vs* control, 1 nM rapamycin, and 10 nM rapamycin.

Scale bar = 50 μm.

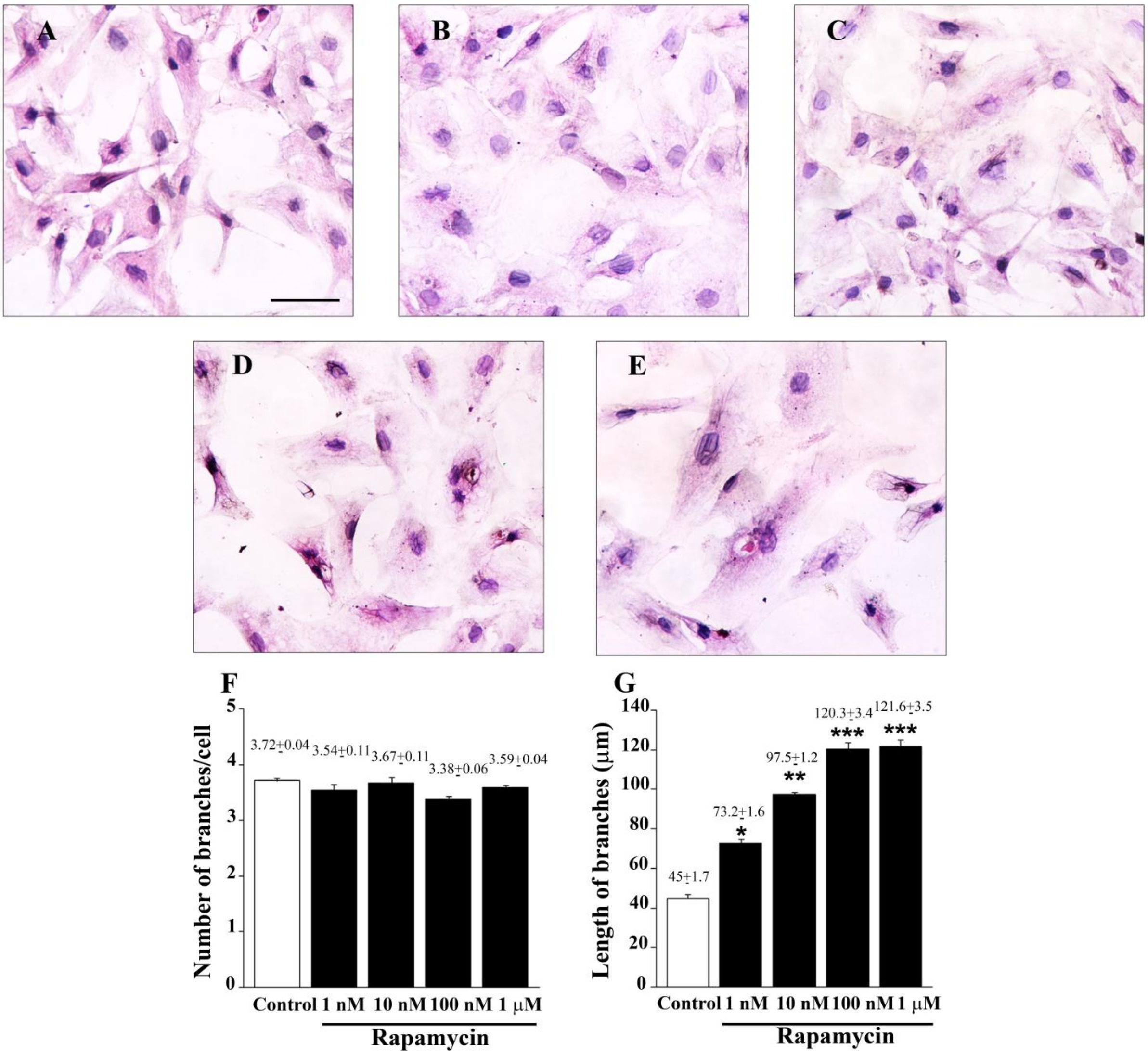

Supplementary Figure 4. Rapamycin dose-dependently increases the length of A172 cell branching.

Representative pictures of H&E-stained A172 cells. The pyramidal or multipolar shape of the cell body in control cells (**A**) is not modified by 1 nM (**B**), 10 nM (**C**), 100 nM (**D**) and 1 μM (**E**) rapamycin, which does not affect the number of cell branching (**F)**. In contrast, the length of cell branches is increased dose-dependently by rapamycin, as reported in graph (**G**).

Values are given as the mean±S.E.M. Comparisons between groups are made by using one-way ANOVA with Scheffé post-hoc test.

*P≤0.05 *vs* control.

**P≤0.05 *vs* control and 1nM rapamycin.

***P≤0.05 *vs* control and rapamycin at 1 nM and 10 nM.

Scale bar = 50 μm.

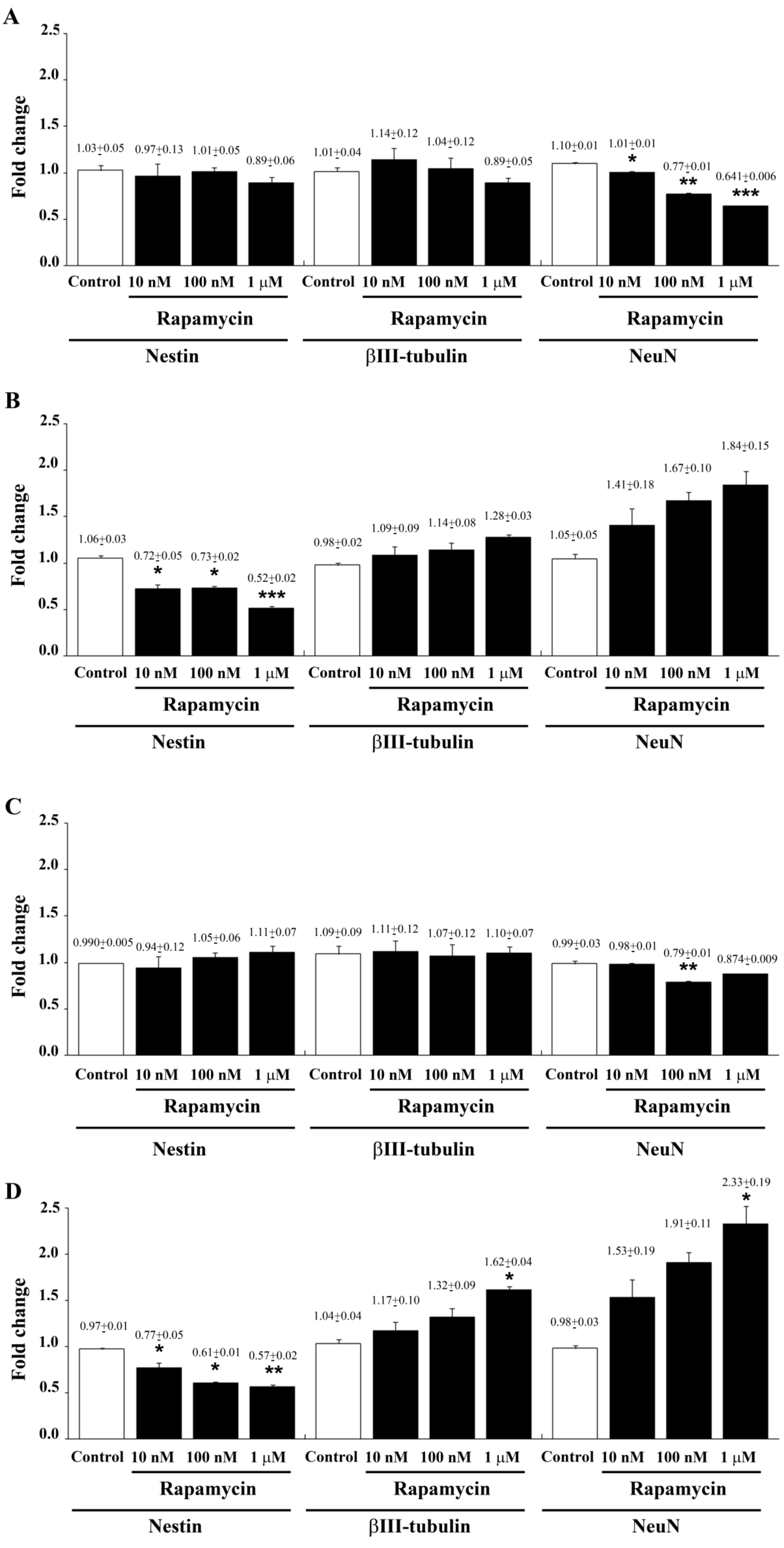
A
B
C
D
Fold change
0.0
0.5
1.0
1.5
2.0
2.5
Control
10 nM
100 nM
1 μM
Rapamycin
Nestin
βIII-tubulin
NeuN
1.03±0.05
0.97±0.13
1.01±0.05
0.89±0.06
1.01±0.04
1.14±0.12
1.04±0.12
0.89±0.05
1.10±0.01
1.01±0.01
0.77±0.01
0.641±0.006
1.06±0.03
0.72±0.05
0.73±0.02
0.52±0.02
0.98±0.02
1.09±0.09
1.14±0.08
1.28±0.03
1.05±0.05
1.41±0.18
1.67±0.10
1.84±0.15
0.990±0.005
0.94±0.12
1.05±0.06
1.11±0.07
1.09±0.09
1.11±0.12
1.07±0.12
1.10±0.07
0.99±0.03
0.98±0.01
0.79±0.01
0.874±0.009
0.97±0.01
0.77±0.05
0.61±0.01
0.57±0.02
1.04±0.04
1.17±0.10
1.32±0.09
1.62±0.04
0.98±0.03
1.53±0.19
1.91±0.11
2.33±0.19

Supplementary Fig. 5. Various internal references mRNAs do not modify the effects of rapamycin on the amount of specific mRNAs.

Gene expression as determined by quantitative real-time PCR analysis for nestin, βIII-tubulin and NeuN in U87MG cells in control conditions and following 4 h (**A**, **C**) and 24 h (**B**, **D**) of 10 nM, 100 nM and 1 μM rapamycin exposure. Data derived from two different experiments are normalized with actin (**A**, **B**) and globulin (**C**, **D**) as internal references.

Data are expressed as the means±S.E.M. Comparison between groups are made by using one-way ANOVA with Bonferroni test.

*P≤0.05 *vs* control.

**P≤0.05 *vs* control and 10 nM rapamycin.

***P≤0.05 *vs* control and rapamycin at 10 nM and 100 nM.

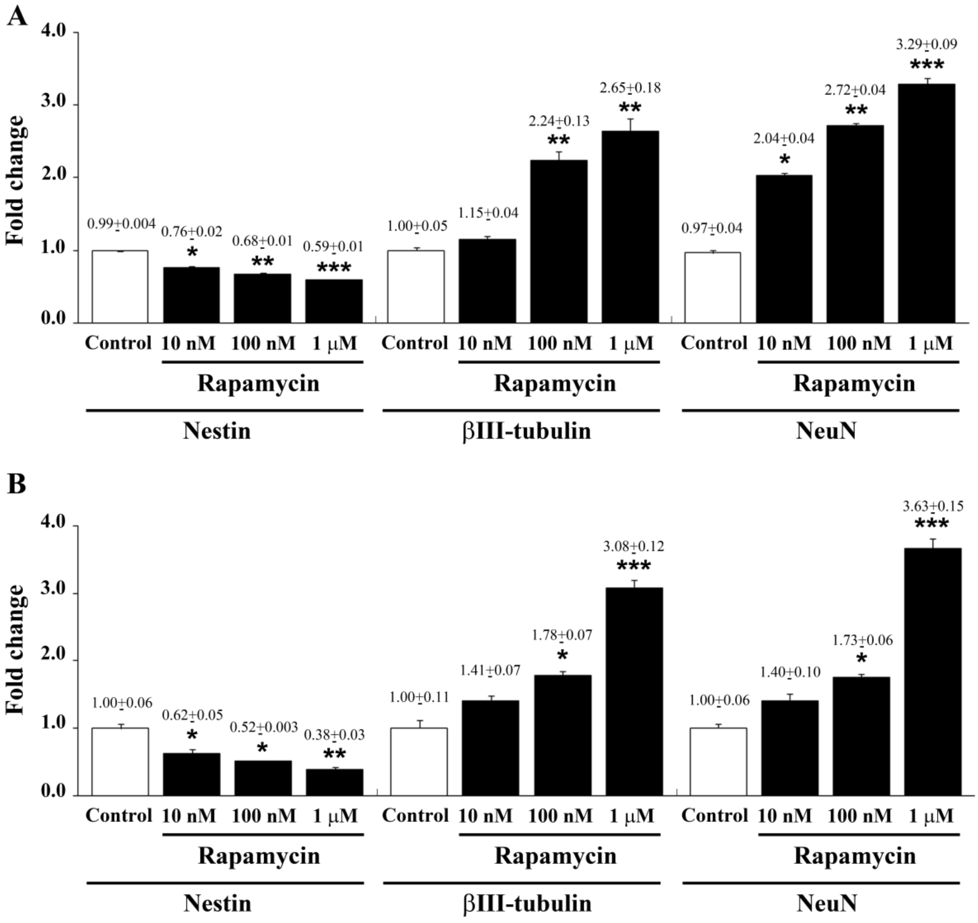

Supplementary Figure 6. Rapamycin dose-dependently reduces nestin and increases βIII-tubulin and NeuN mRNAs in both U251MG and A172 cell lines.

Nestin, βIII-tubulin and NeuN gene expression (reported as the means from three different experiments normalized with two different internal references, globin and actin, by quantitative real-time PCR analysis) in U251MG cells (**A**) and A172 cells (**B**) are shown, in control conditions and following 24 h of 10 nM, 100 nM and 1 μM rapamycin.

Data are expressed as the means±SEM. Comparison between groups are made by using one-way ANOVA with Bonferroni test.

*P≤0.05 *vs* control.

**P≤0.05 *vs* control and 10 nM rapamycin.

***P ≤ 0.05 *vs* control and rapamycin at 10 nM and 100 nM.

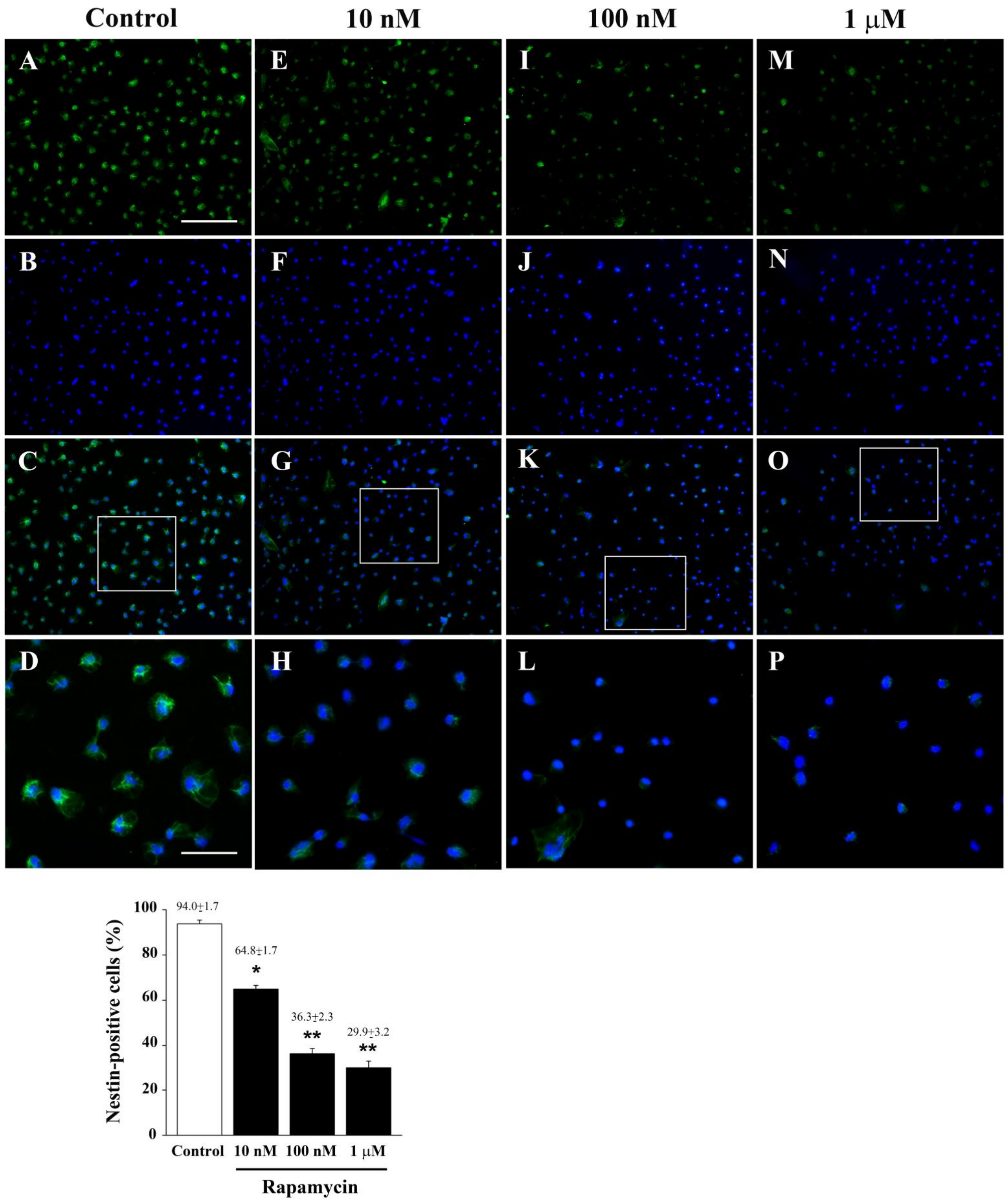

Supplementary Figure 7. Rapamycin dose-dependently decreases nestin in U251MG cells. Immune-fluorescence of U251MG cells treated either with vehicle (**A-D**) or rapamycin at the dose of 10 nM (**E-H**); 100 nM (**I-L**); 1 μM (**M-P**). In the first line cells were stained for the stem cell marker nestin. Rapamycin decreases dose-dependently nestin immune-fluorescence. In the second

line each group of cells was stained for the nuclear dye DAPI. In the third line the merging between nestin (green) and DAPI (blue) fluorescence is shown. In the fourth line, high magnification of the squared insert of line three is shown. The graph reports the percentage of nestin-positive cells in control and after treatment with different doses of rapamycin.

Values are given as the mean±S.E.M. Comparisons between groups are made by using one-way ANOVA with Scheffé post-hoc test.

*$P \leq 0.05$ *vs* control.

**$P \leq 0.05$ *vs* control and 10 nM rapamycin.

Scale bars = (**A**-**C**, **E**-**G**, **I**-**K**, **M**-**O**) 155 μm; (**D**, **H**, **L**, **P**) 45 μm.

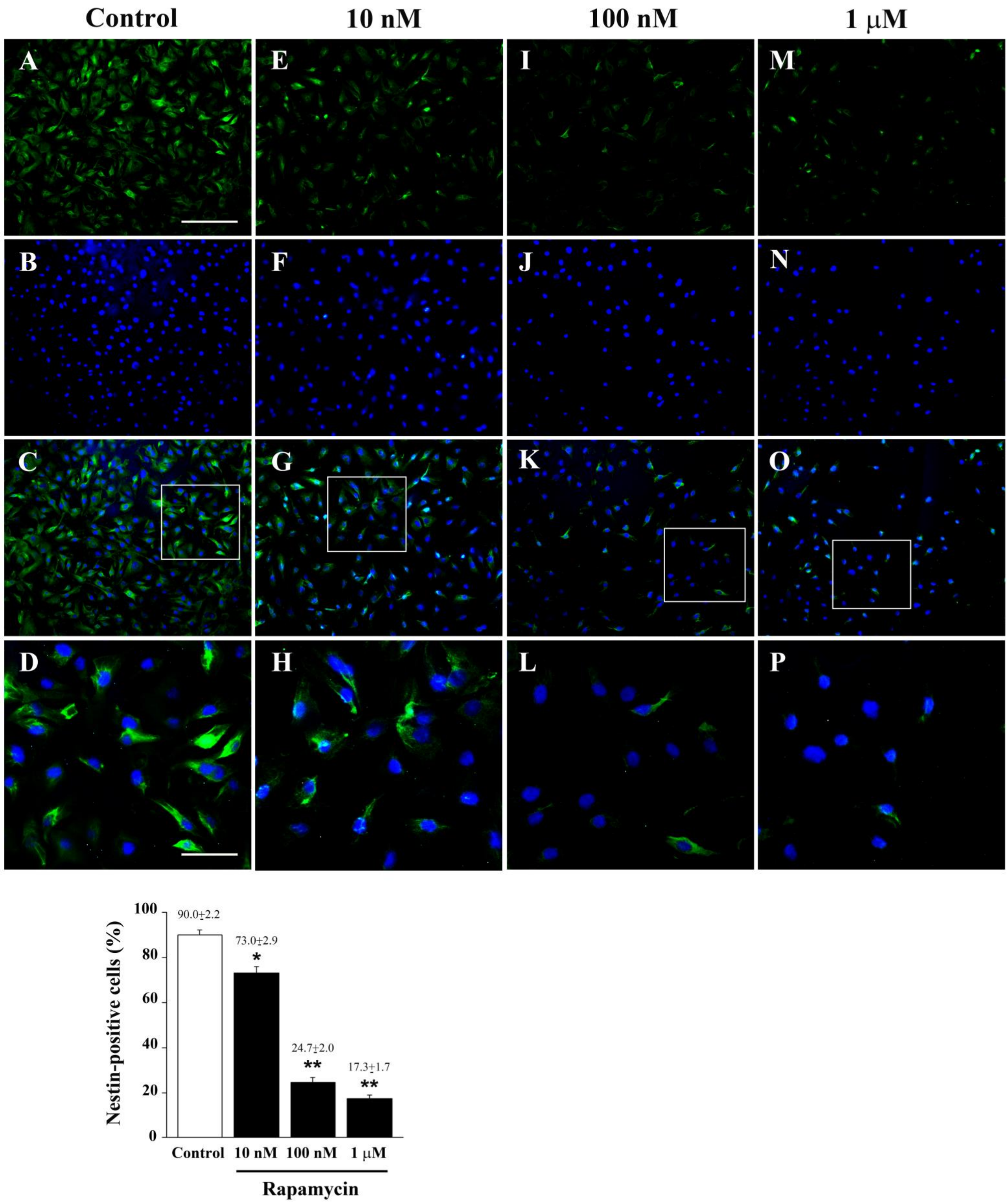

Supplementary Figure 8. Rapamycin dose-dependently reduces nestin in A172 cells.

Immune-fluorescence of A172 cells treated either with vehicle(**A-D**) or rapamycin at the dose of 10 nM (**E-H**); 100 nM (**I-L**) and 1 μM (**M-P**). In the first line cells were stained for the stem cell marker nestin. Rapamycin decreases dose-dependently nestin immune-fluorescence. In the second

line cells were stained for the nuclear dye DAPI. In the third line the merging between nestin (green) and DAPI (blue) fluorescence is shown. In the fourth line, a high magnification of the squared insert of line three is shown. The graph reports the percentage of nestin-positive cells in control and after treatment with different doses of rapamycin.

Values are given as the mean±S.E.M. Comparisons between groups are made by using one-way ANOVA with Scheffé post-hoc test.

*P≤0.05 *vs* control.

**P≤0.05 *vs* control and 10 nM rapamycin.

Scale bars = (**A**-**C**, **E**-**G**, **I**-**K**, **M**-**O**) 155 μm; (**D**, **H**, **L**, **P**) 45 μm.

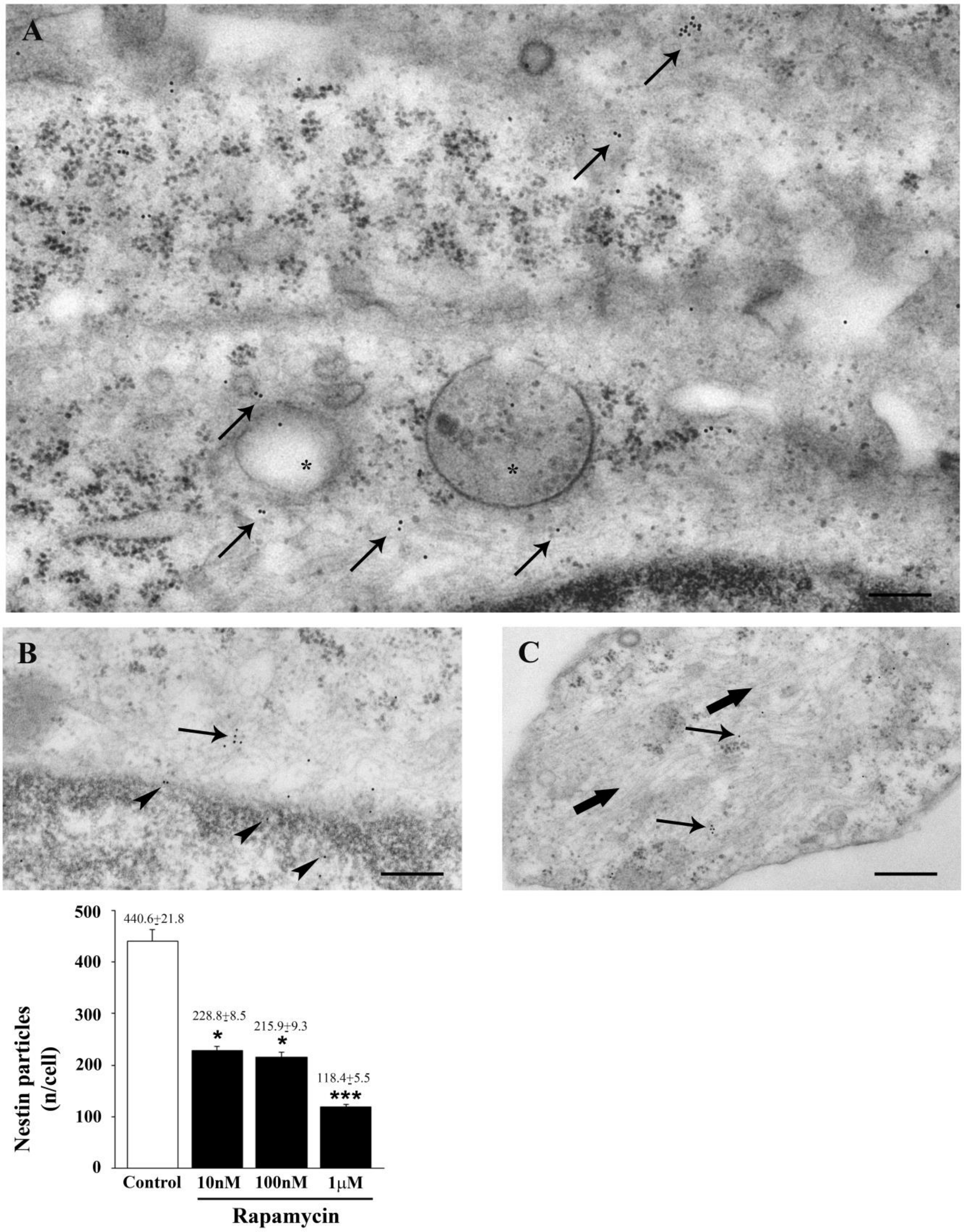

Supplementary Figure 9. Rapamycin dose-dependently reduces nestin immune-cytochemistry in U251MG cells.

Representative micrographs of immune-citochemistry for nestin in control cells (**A**-**C**). Nestin immune-gold particles are localized in the cytoplasm (arrows) and autophagy-like vacuoles (*) (**A**). Nestin immune-gold particles (arrow) are present in the cytoplasm and nucleus (arrowheads) (**B**). In Immune-gold particles (arrows) are present on filaments of a cell branch (thick arrows) (**C**). The

graph reports counts of nestin immune-gold which are reduced dose-dependently by rapamycin (10 nM; 100 nM and 1 μM).

Values are given as the mean±S.E.M. Comparisons between groups are made by using one-way ANOVA with Scheffé post-hoc test.

*P ≤ 0.0001 *vs* control.

***P ≤ 0.0001 *vs* control and rapamycin at 10 nM and 100 nM.

Scale bars= (**A**) = 0.19 μm; (**B**,**C**) = 0.45 μm.

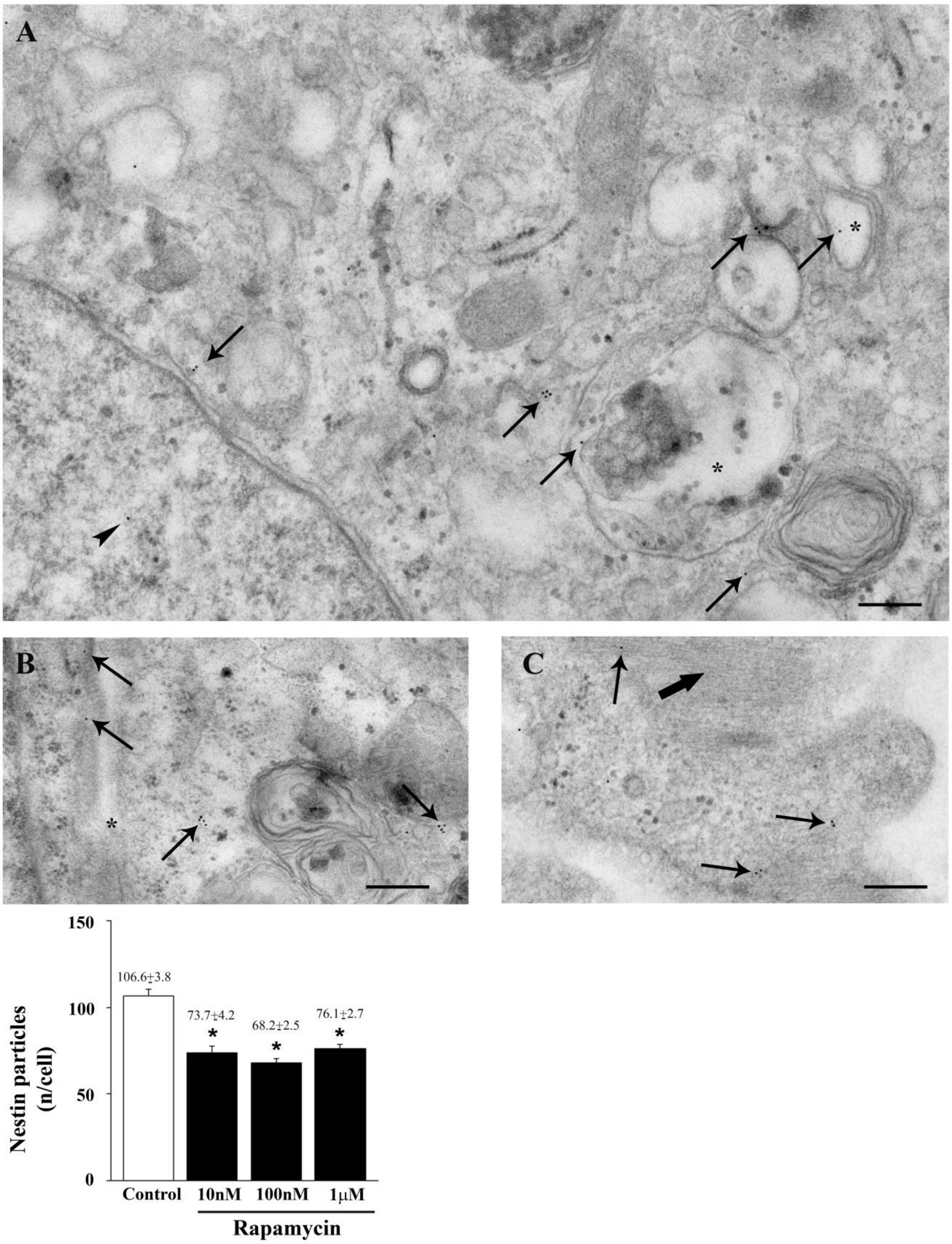

Supplementary Figure 10. Rapamycin dose-dependently reduces nestin immune-cytochemistry in A172 cells.

Representative pictures of immune-cytochemistry for nestin in control cells (**A**-**C**). Nestin immune-gold particles are localized in the cytoplasm (arrows), autophagy-like vacuoles (*) and nucleus (arrowhead) (**A**). Clusters of nestin immune-gold particles (arrows) are evident in the cytoplasm and within a narrow space between two cells (*) (**B**). Immune-gold particles are evident on filaments

(thick arrow) of a cell branch (arrows) (**C**). The graph reports the rapamycin-induced dose-dependent decrease of nestin immune-gold particles.

Values are given as the mean±S.E.M. Comparisons between groups are made by using one-way ANOVA with Scheffé post-hoc test.

*P ≤ 0.0001 *vs* control

Scale bars= (**A**) = 0.39 μm; **(B**,**C**) = 0.5 μm.

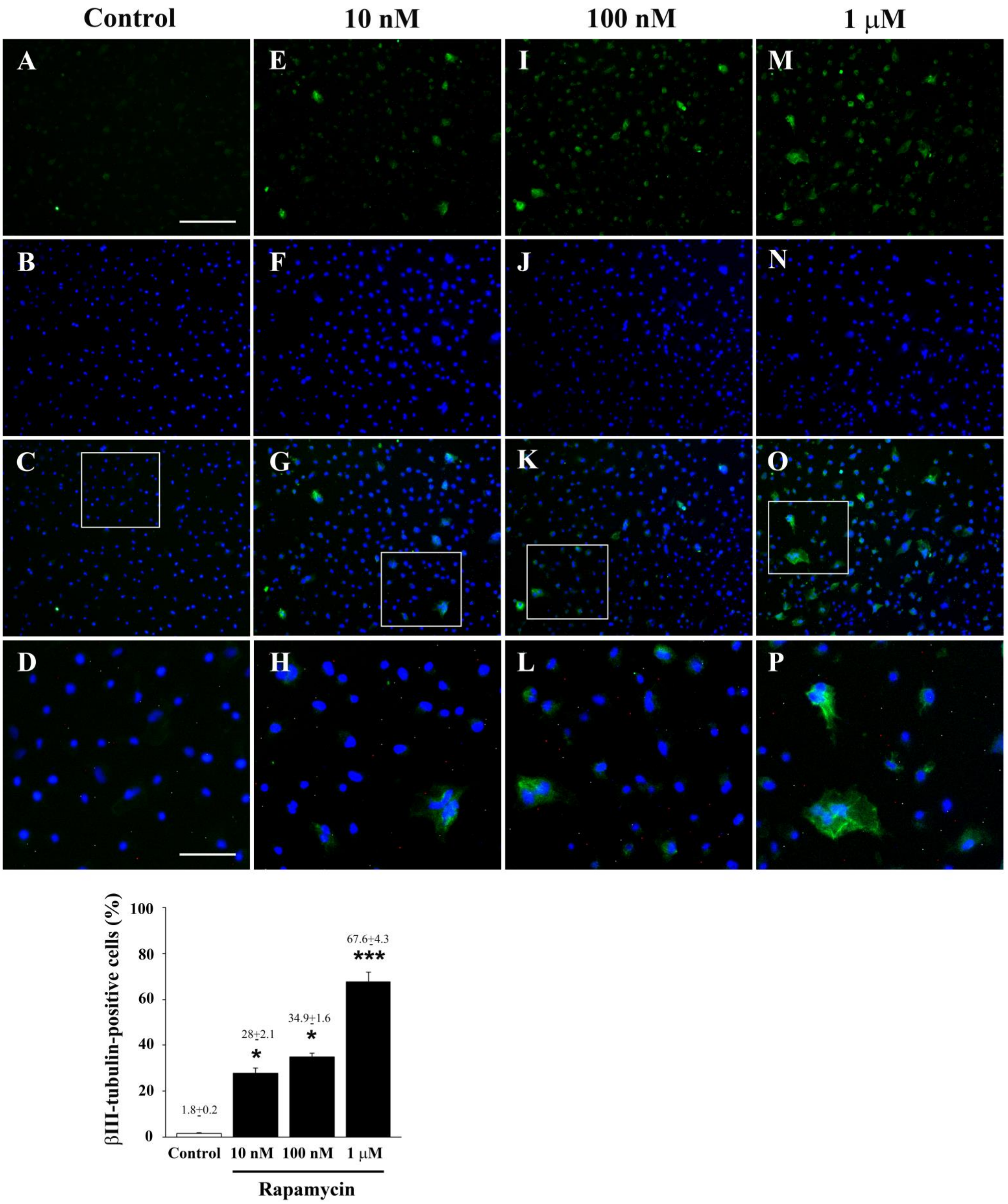

Supplementary Figure 11. Rapamycin dose-dependently increases βIII-tubulin immune-fluorescence in U251MG cells.

Immune-fluorescence of U251MG cells treated with vehicle (**A-D**) and rapamycin: 10 nM (**E-H**); 100 nM (**I-L**); 1 μM (**M-P**). In the first line cells were stained for the early neuronal marker βIII-

tubulin. Rapamycin increases dose-dependently βIII-tubulin immune-fluorescence. In the second line cells were stained for the nuclear dye DAPI. In the third line the merging between βIII-tubulin (green) and DAPI (blue) fluorescence is shown.

In the fourth line a high magnification of the squared insert of line three is reported. The graph reports the percentage of βIII-tubulin-positive cells in control and after treatment with different doses of rapamycin.

Values are given as the mean±S.E.M. Comparisons between groups were made by using one-way ANOVA with Scheffé post-hoc test.

*P≤0.05 *vs* control.

***P≤0.05 *vs* control and rapamycin at 10 nM and 100 nM.

Scale bars = (**A**-**C**, **E**-**G**, **I**-**K**, **M**-**O**) 155 μm; (**D**, **H**, **L**, **P**) 45 μm.

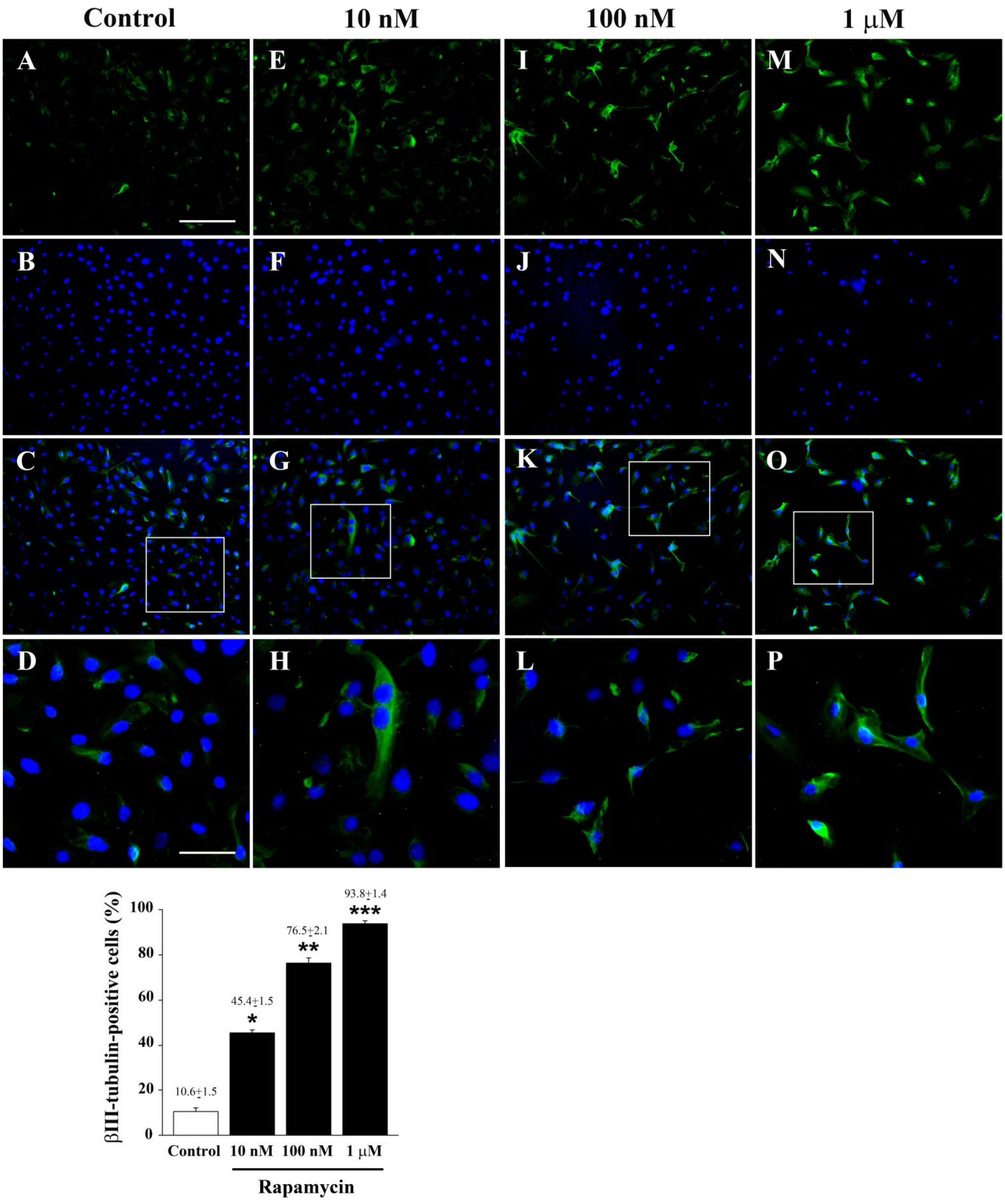

Supplementary Figure 12. Rapamycin dose-dependently increases βIII-tubulin immune-fluorescence in A172 cells.

Immune-fluorescence of A172 cells treated with vehicle (**A-D**) and rapamycin: 10 nM (**E-H**), 100 nM (**I-L**), and 1 μM (**M-P**). In the first line cells were stained for the early neuronal marker βIII-

tubulin. Rapamycin increases dose-dependently βIII-tubulin immune-fluorescence. In the second line cells were stained for the nuclear dye DAPI. In the third line the merging between βIII-tubulin (green) and DAPI (blue) fluorescence is shown.

In the fourth line a high magnification of the squared insert of line three is reported. The graph reports the percentage of βIII-tubulin-positive cells in control and after treatment with different doses of rapamycin.

Values are given as the mean±S.E.M. Comparisons between groups were made by using one-way ANOVA with Scheffé post-hoc test.

*P≤0.05 *vs* control.

**P≤0.05 *vs* control and 10 nM rapamycin.

***P≤0.05 *vs* control and rapamycin at 10 nM and 100 nM.

Scale bars = (**A-C**, **E-G**, **I-K**, **M-O**) 155 μm; (**D**, **H**, **L**, **P**) 45 μm.

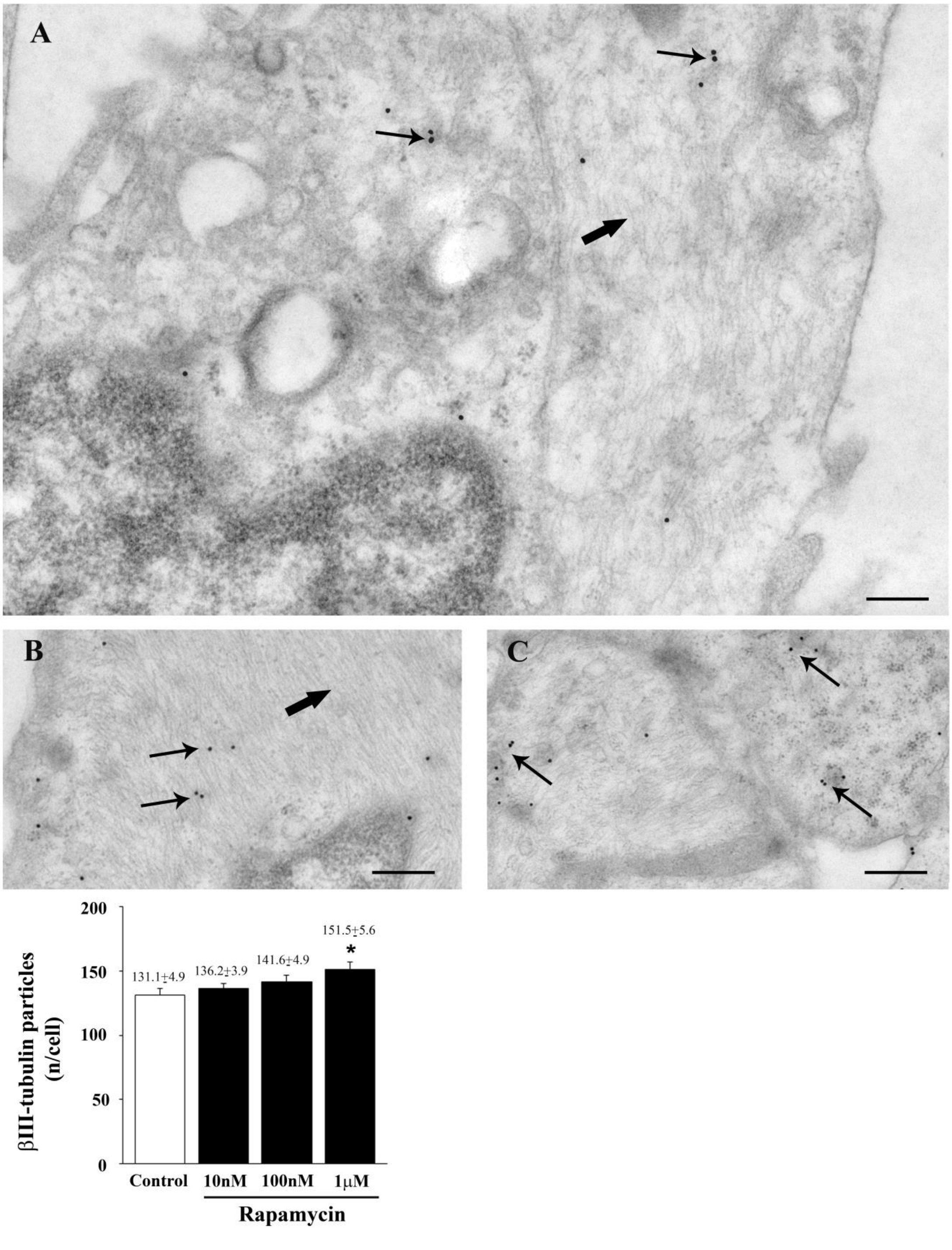

Supplementary Figure 13. Rapamycin increases βIII-tubulin immune-cytochemistry in U251MG cells.

In representative micrograph (**A**) scanty immune-gold particles (arrows) of βIII-tubulin in the cytoplasm and filaments (thick arrow) of a control cell are shown. After rapamycin immune-gold particles (arrows) dose-dependently increase and they are localized close to cytoplasmic filaments

(thick arrow) (**B**) and within cell branches (arrows) (**C**). The graph reports the quantitative effects of rapamycin (10 nM; 100 nM and 1 μM)-induced increase in βIII-tubulin.

Values are given as the mean±S.E.M. Comparisons between groups were made by using one-way ANOVA with Scheffé post-hoc test.

*P ≤ 0.05 *vs* control.

Scale bars= (**A**) = 0.42 μm; (**B**, **C**) = 0.63 μm.

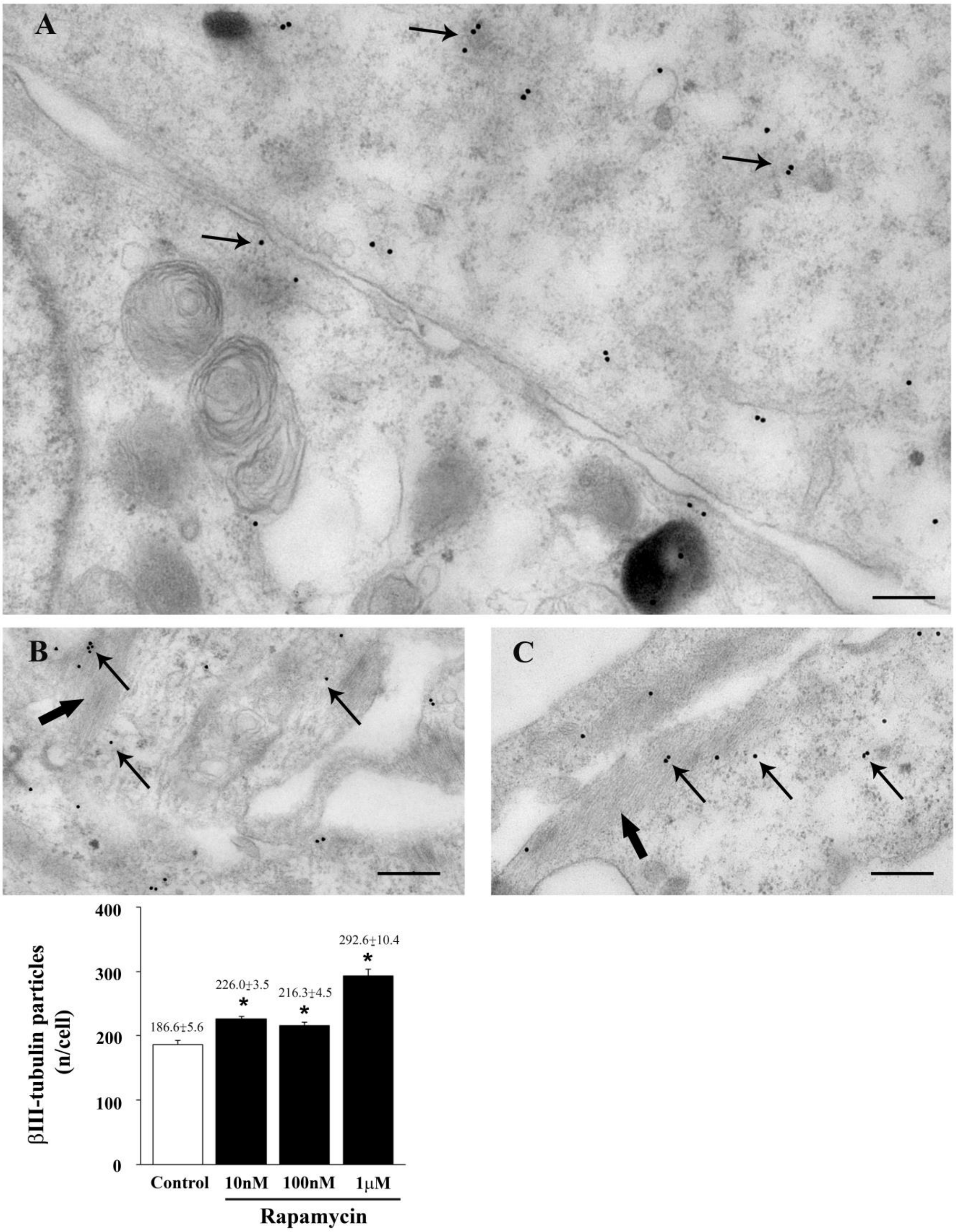

Supplementary Figure 14. Rapamycin increases βIII-tubulin immune-cytochemistry in A172 cells.

In representative micrograph (**A**) scattered immune-gold particles (arrows) of βIII-tubulin are shown in the cytoplasm of two control cells. After rapamycin (**B**, **C**) immune-gold particles (arrows) are more abundant and possess a more specific localization close to filaments (thick arrows), in the cytoplasm (**B**) and within cell branches (**C**). The graph reports the dose-dependent increase of βIII-tubulin induced by rapamycin.

Values are given as the mean±S.E.M. Comparisons between groups were made by using one-way ANOVA with Scheffé post-hoc test.

*$P \leq 0.001$ *vs* control.

Scale bars= (**A**)= 0.4 μm; (**B**) = 0.45 μm; **(C**) = 0.6 μm.

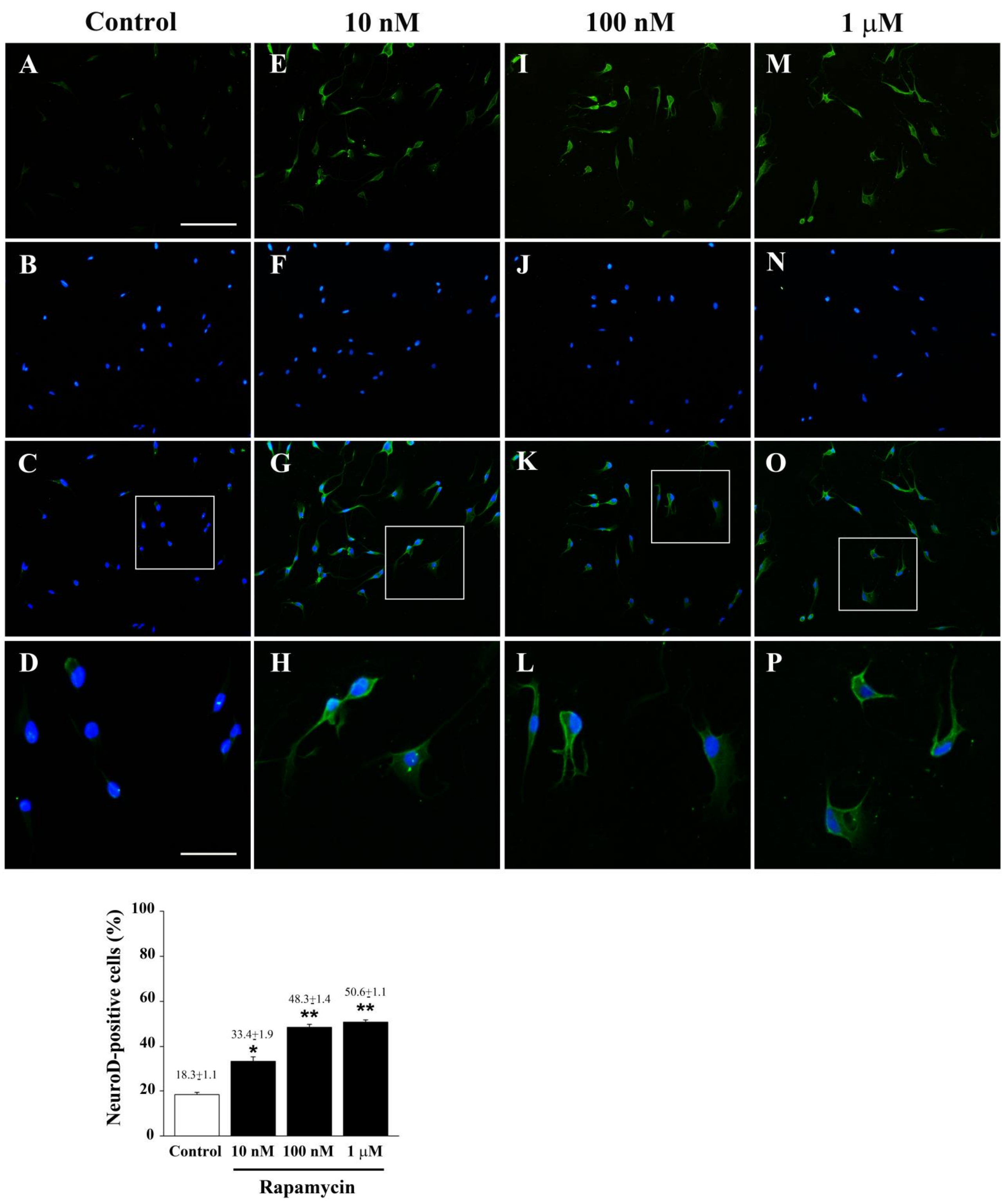

Supplementary Figure 15. Rapamycin dose-dependently increases NeuroD immune-fluorescence in U87MG cells.

Immune-fluorescence of U87MG cells treated either with vehicle (**A-D**) or rapamycin at the dose of 10 nM (**E-H**); 100 nM (**I-L**); 1 μM (**M-P**). In the first line cells were stained for the early neuronal

marker NeuroD. Rapamycin increases NeuroD immune-fluorescence. In the second line cells were stained for the nuclear dye DAPI. In the third line the merging between NeuroD (green) and DAPI (blue) fluorescence is shown.

In the fourth line a high magnification of the squared insert of line three is shown. The graph reports NeuroD positive cells in control and after treatment with different doses of rapamycin.

Values are given as the mean±S.E.M. Comparisons between groups are made by using one-way ANOVA with Scheffé post-hoc test.

*$P \leq 0.05$ *vs* control.

**$P \leq 0.05$ *vs* control and 10 nM rapamycin.

Scale bars = (**A**-**C**, **E**-**G**, **I**-**K**, **M**-**O**) 155 μm; (**D**, **H**, **L**, **P**) 45 μm.

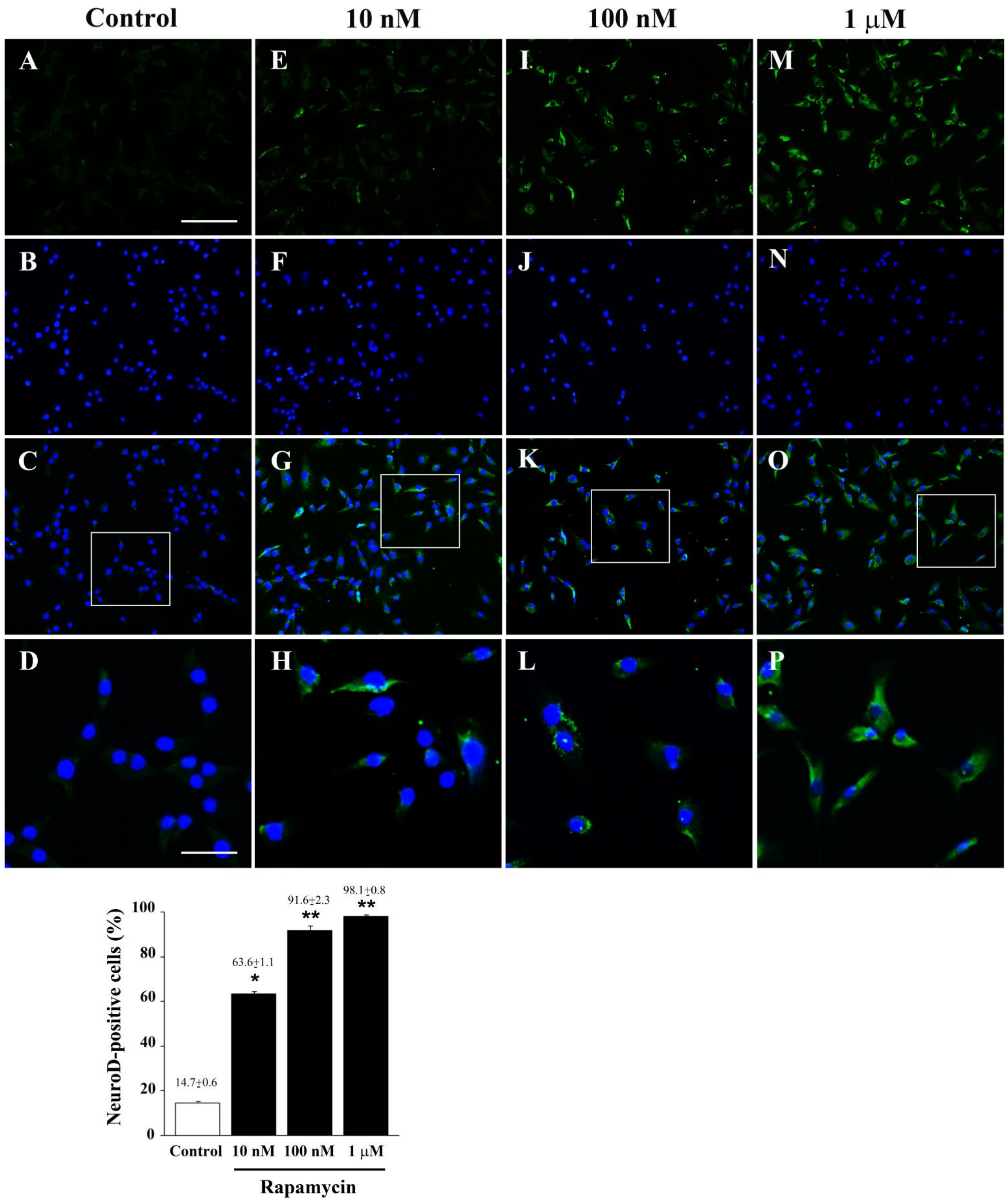

Supplementary Figure 16. Rapamycin dose-dependently increases NeuroD immune-fluorescence in A172 cells.

Immune-fluorescence of A172 cells treated either with vehicle (**A-D**) or rapamycin at the dose of 10 nM (**E-H**); 100 nM (**I-L**); 1 μM (**M-P**). In the first line cells were stained for the early neuronal

marker NeuroD. Rapamycin increases NeuroD immune-fluorescence. In the second line cells were stained for the nuclear dye DAPI. In the third line the merging between NeuroD (green) and DAPI (blue) fluorescence is shown. In the fourth line a high magnification of the squared insert of line three is shown. The graph reports NeuroD positive cells in control and after treatment with different doses of rapamycin.

Values are given as the mean±S.E.M. Comparisons between groups are made by using one-way ANOVA withScheffé post-hoc test.

*$P \leq 0.05$ *vs* control.

**$P \leq 0.05$ *vs* control and 10 nM rapamycin.

Scale bars = (**A**-**C**, **E**-**G**, **I**-**K**, **M**-**O**) 155 μm; (**D**, **H**, **L**, **P**) 45 μm.

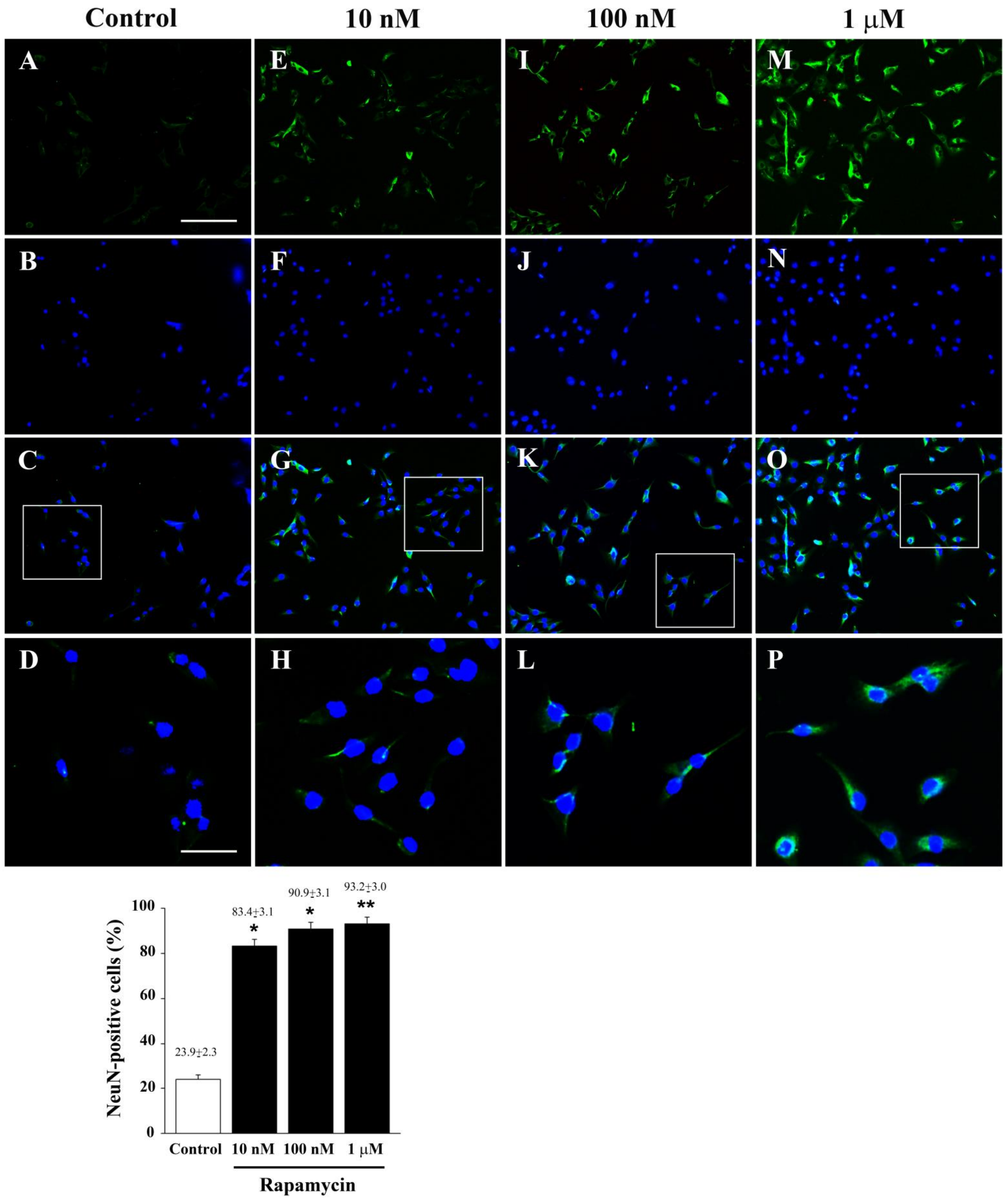

Supplementary Figure 17. Rapamycin dose-dependently increases NeuN immune-fluorescence in A172 cells.

Immune-fluorescence of A172 cells treated either with vehicle (**A-D**) or rapamycin at the dose of 10 nM (**E-H**), 100 nM (**I-L**), an 1 μM (**M-P**) is shown. In the first line each group of cells was stained

for the late post-mitotic neuronal marker NeuN. Rapamycin increases dose-dependently NeuN immune-fluorescence. In the second line, each group of cells was stained for the nuclear dye DAPI. In the third line the merging between NeuN (green) and DAPI (blue) fluorescence is shown. In the fourth line a high magnification of the squared insert of line three is shown. The graph reports the percentage of NeuN-positive cells in control and after treatment with different doses of rapamycin. Values are given as the mean±S.E.M. Comparisons between groups were made by using one-way ANOVA with Scheffé post-hoc test.

*P≤0.05 *vs* control.

**P≤0.05 *vs* control and 10 nM rapamycin.

Scale bars = (**A**-**C**, **E**-**G**, **I**-**K**, **M**-**O**) 155 μm; (**D**, **H**, **L**, **P**) 45 μm.

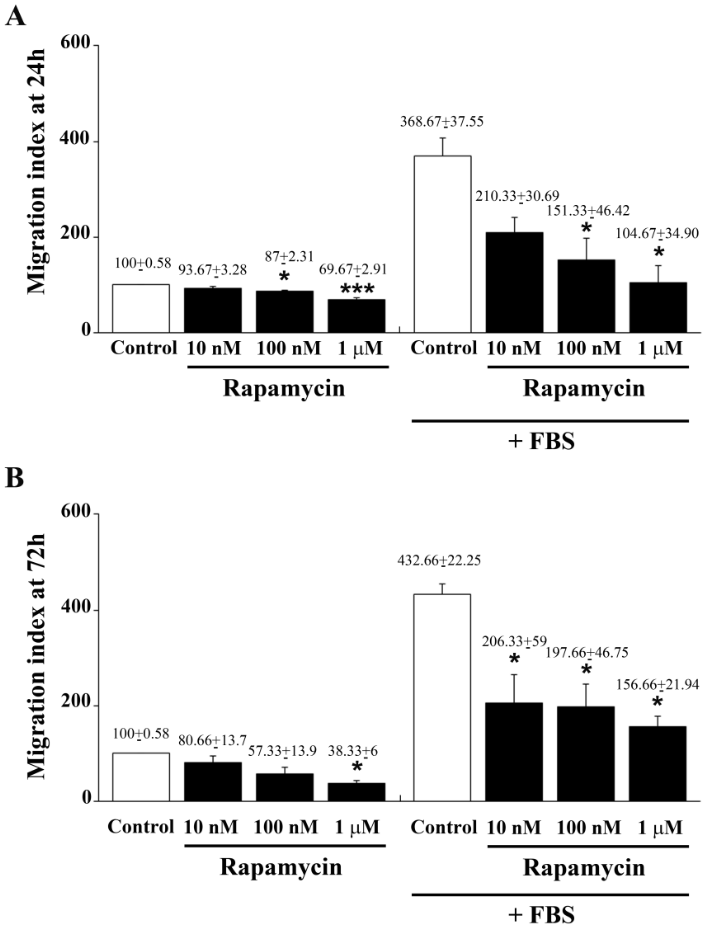

Supplementary Figure 18. Rapamycin dose-dependently suppresses FBS-induced U251MG cell migration.

Graphs report the migration index of U251MG cells towards a medium with or without FBS for 3h. Cells were treated either 24 h (**A**) or 72 h (**B**) with different doses of rapamycin. Data are expressed as percentage of migrated cells in comparison with control without FBS at 72 h (taken as 100%). Comparisons between groups were made by using one-way ANOVA with Scheffé post-hoc test.

*P≤0.05 *vs* respective control.

***P≤0.05 *vs* respective control and rapamycin at 10 nM and 100 nM.

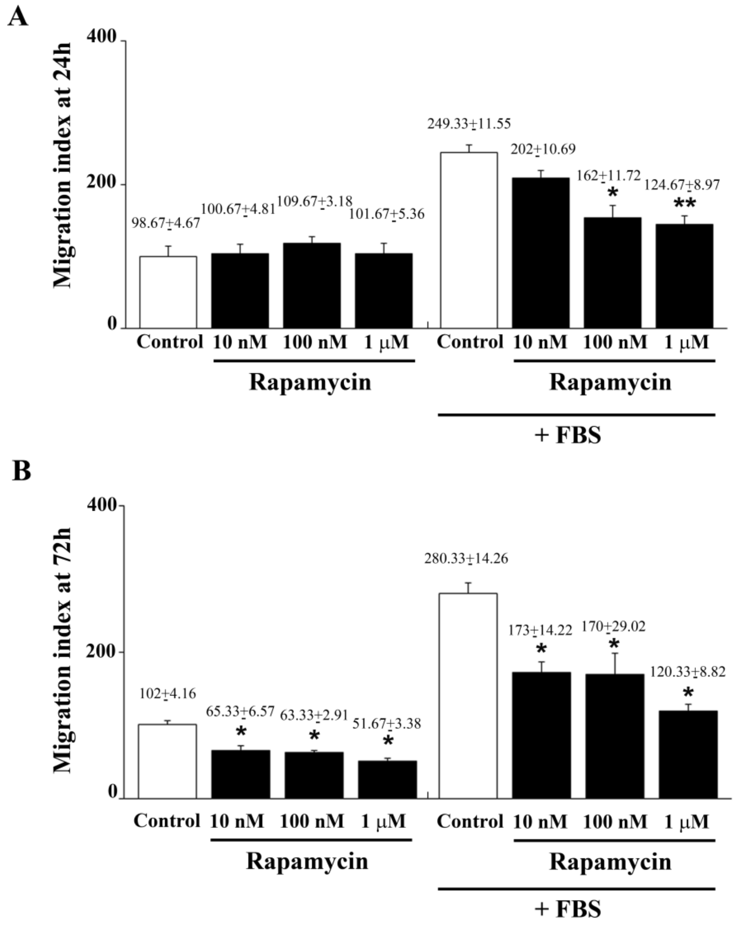

Supplementary Figure 19. Rapamycin dose-dependently suppresses FBS-induced A172 cell migration.

Graphs report the migration index of A172 cells towards a medium with or without FBS for 3h. Cells were treated either 24 h (**A**) or 72 h (**B**) with different doses of rapamycin. Data are expressed as percentage of migrated cells in comparison with the total control without FBS (24 h and 72 h, taken as 100%).

Comparisons between groups were made by using one-way ANOVA with Scheffé post-hoc test.

*P≤0.05 *vs* respective control.

**P≤0.05 *vs* respective control and 10 nM rapamycin.

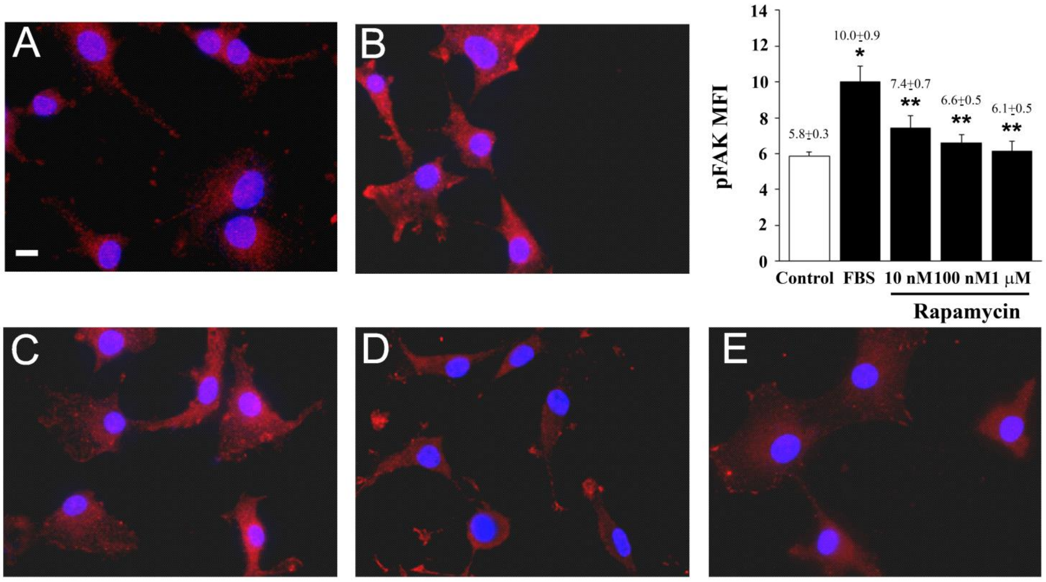

Supplementary Figure 20. Rapamycin dose-dependently suppresses pFAK immune-fluorescence in U87MG cells.

U87MG treated with different concentrations of rapamycin were assayed for pFAK by immune-fluorescence. Representative pictures of non-treated cells (**A**); FBS-treated cells in the absence (**B**) or in the presence of 10 nM (**C**), 100 nM (**D**) or 1 µM (**E**) rapamycin (pFAK in red, Hoechst in blue). Data in the graph are the mean fluorescence intensity (MFI)±S.E.M. Comparisons between groups were made by using one-way ANOVA.

*P≤0.05 *vs* control.

**P≤0.05 *vs* FBS.

Scale bar = 20 µm.

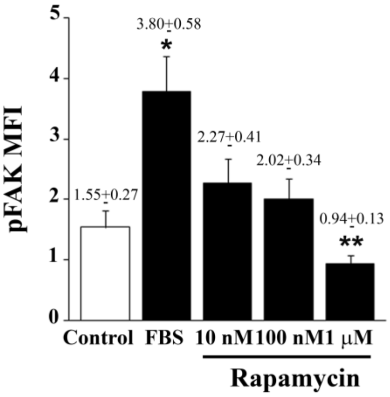

Supplementary Figure 21. Rapamycin dose-dependently suppresses pFAK immune-fluorescence in the U251MG cell line.

U251MG treated with different concentrations of rapamycin were assayed for pFAK by immune-fluorescence. Graph reports the mean fluorescence intensity for pFAK, expressed as (MFI)±S.E.M., of non-treated cells (control), FBS-treated cells in the absence and in the presence of rapamycin (10 nM, 100 nM and 1 μM).

Comparisons between groups were made by using one-way ANOVAwith Scheffé post-hoc test.

*P≤0.05 *vs* control.

**P≤0.05 *vs* FBS.

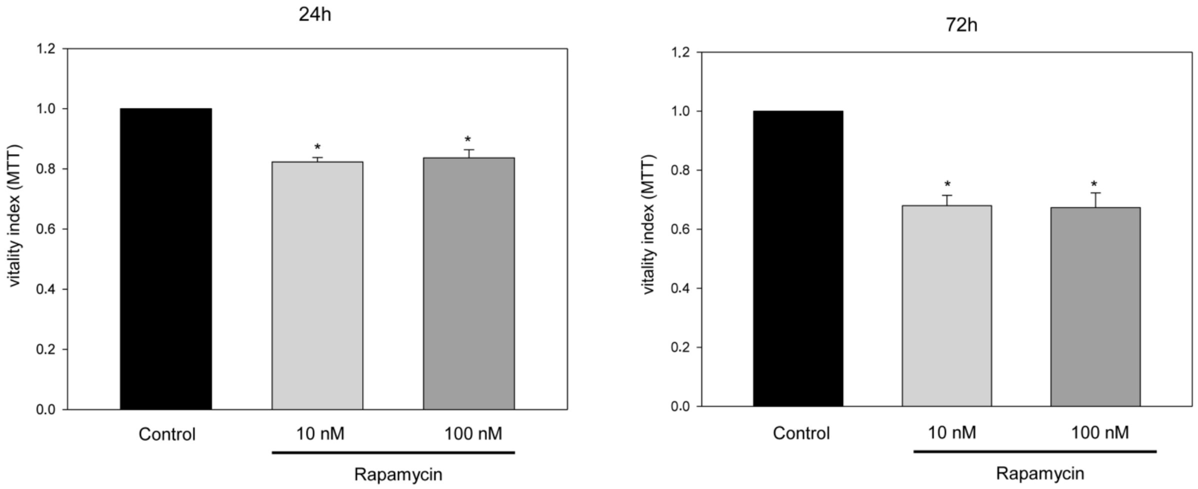

Supplementary Figure 22. U87MG cell viability is slightly decreased by rapamycin.

The measurement of cell viability with the MTT assay was carried out as a pilot study preliminarily to assess cell migration. The assay demonstrates that rapamycin slightly decreases cell viability, which was also reported in a previous study we published [23]. This effects is much more evident at 72 h than 24 h.

Values are given as the mean±S.E.M. Comparisons between groups are made by using one-way ANOVA with Scheffé post-hoc test.

*P≤0.05 *vs* control.

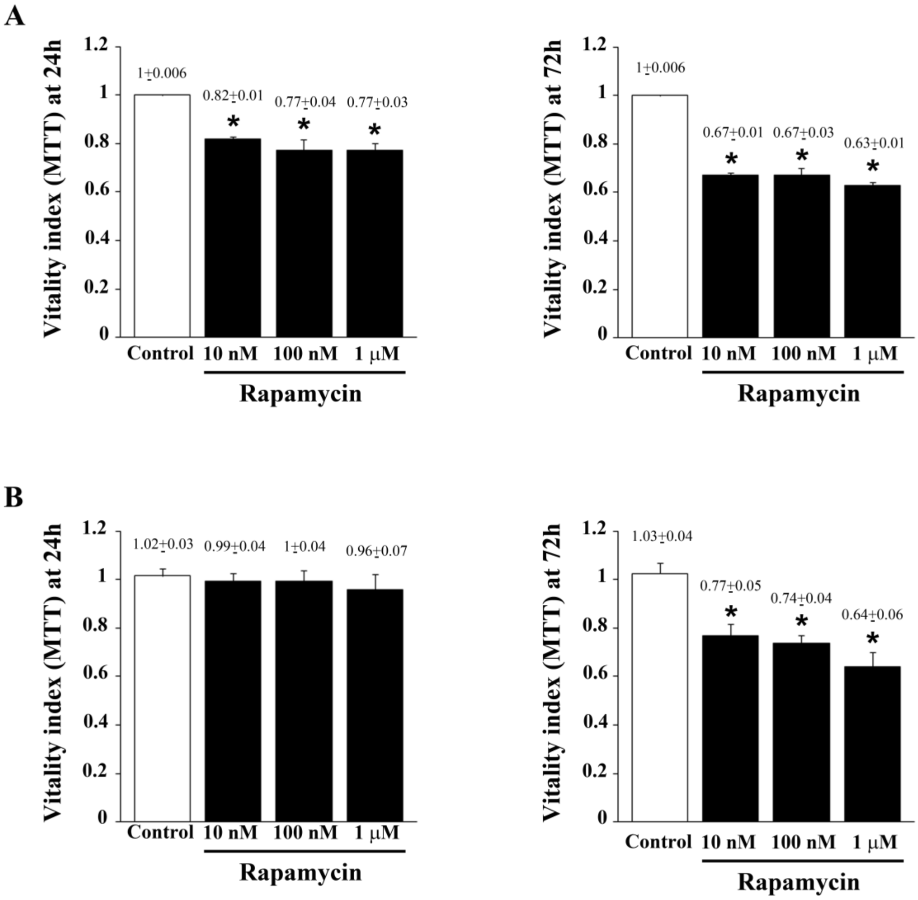

Supplementary Figure 23. Rapamycin slightly decreases cell viability in U251MG and A172 cell lines.

The measurement of cell viability with the MTT assay demonstrates that rapamycin slightly decreases cell viability in U251MG (**A**) and A172 (**B**) cells. This effect is well evident at 72 h in both the cell lines, but it is also present at 24 h in U251MG cells.

Values are given as the mean±S.E.M. Comparisons between groups are made by using one-way ANOVA with Scheffé post-hoc test.

*P≤0.05 *vs* control.

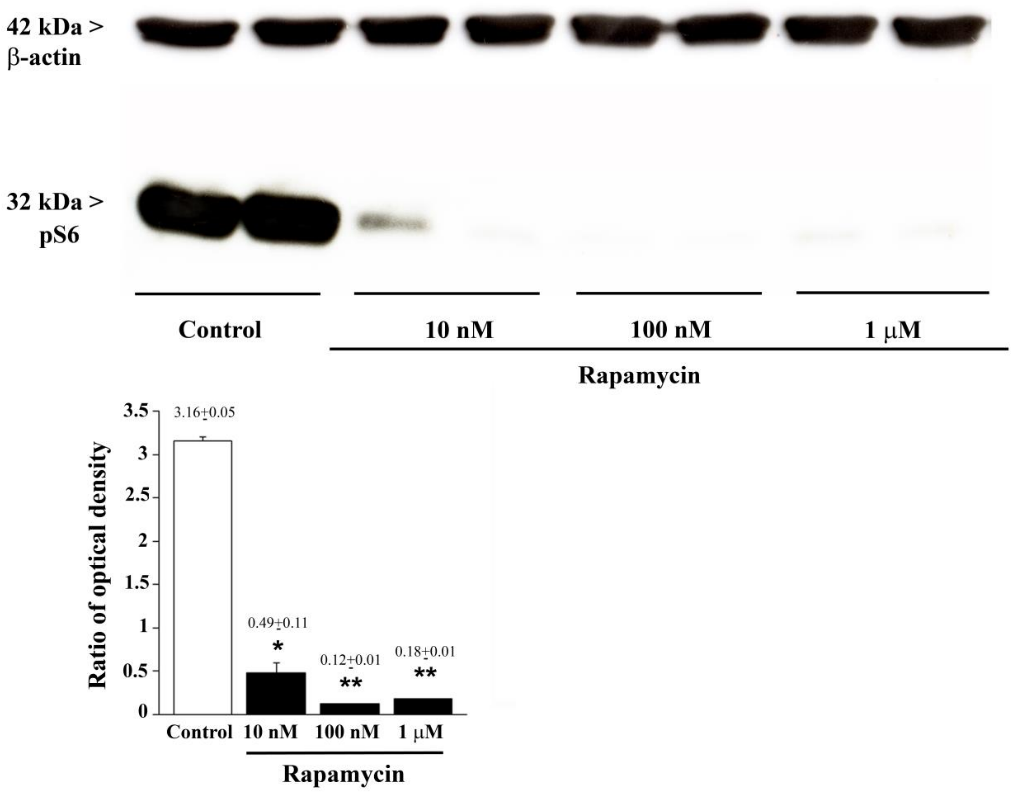

Supplementary Figure 24. Rapamycin dose-dependently reduces p6S in the U87MG cell line.

Representative immune-blotting for the ribosomal protein p6S and the house keeping protein β-actin in control and rapamycin-treated U87MG cells. The ratio between optical densities of p6S and β-actin is reported in the graph. Rapamycin induces a dose-dependent reduction of the mTOR downstream product p6S.

Values are given as the mean±S.E.M. Comparisons between groups were made by using one-way ANOVA with Bonferroni test.

*P≤0.05 *vs* control.

**P≤0.05 *vs* control and 10 nM rapamycin.

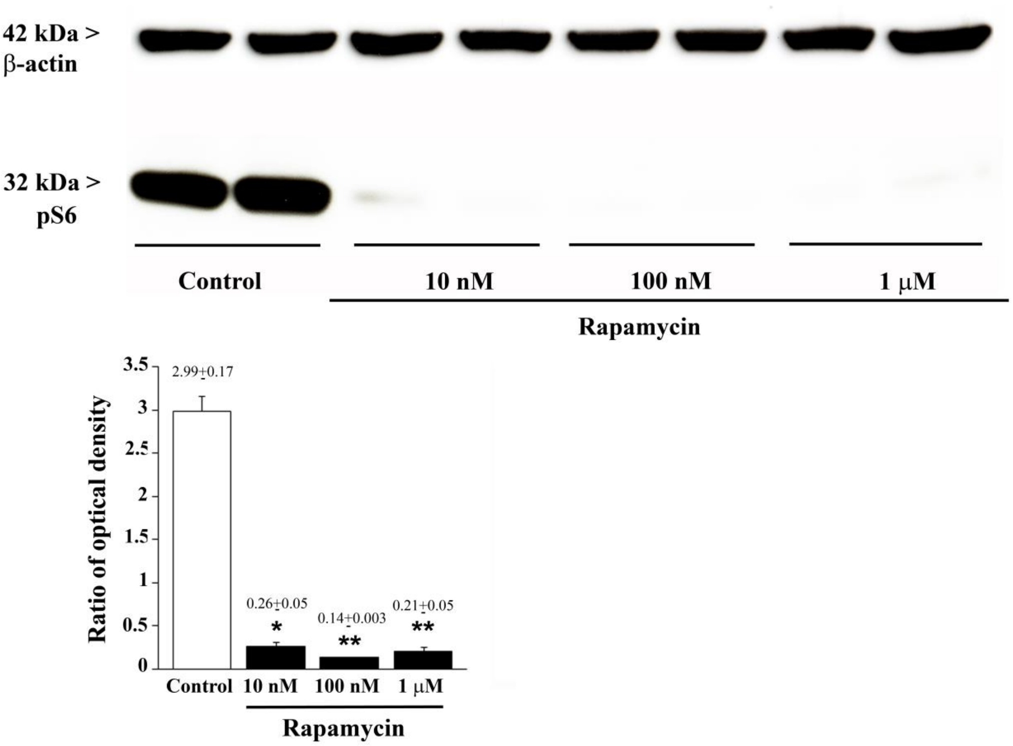

Supplementary Figure 25. Rapamycin dose-dependently reduces p6S immune-blotting in the U251MG cell line.

Representative immune-blotting for the ribosomal protein p6S and the housekeeping protein β-actin in control and rapamycin-treated U251MG cells. The ratio between the optical densities of p6S and β-actin is reported in the graph. Rapamycin induces a dose-dependent reduction of p6S.

Values are given as the mean±S.E.M. Comparisons between groups were made by using one-way ANOVA with Bonferroni test.

*P≤0.05 *vs* control.

**P≤0.05 *vs* control and 10 nM rapamycin.

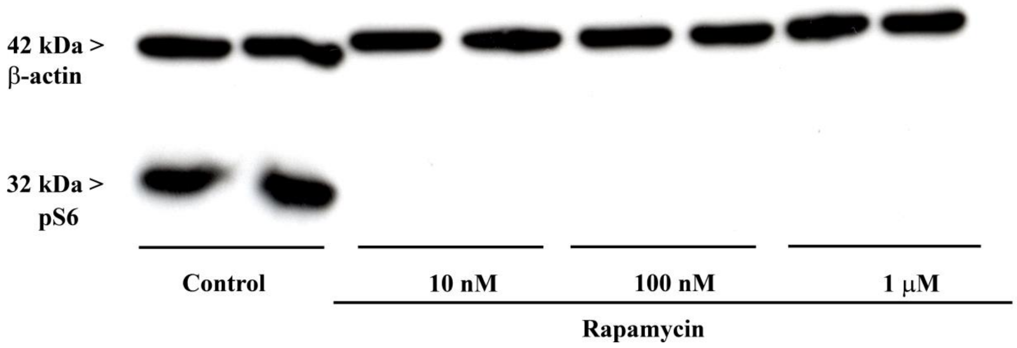

Supplementary Figure 26. Rapamycin dose-dependently reduces p6S immune-blotting in the A172 cell line.

Representative immune-blotting for the ribosomal protein p6S and the housekeeping protein β-actin in control and rapamycin-treated A172 cells. The severe suppression of p6S blotting induced by rapamycin in this cell line did not allow to detect any optical density for the ribosomal protein p6S following rapamycin even at the lowest dose (see results).

No inferential statistics was necessary in this experiment.
